# The genome of the Pyrenean desman and the effects of bottlenecks and inbreeding on the genomic landscape of an endangered species

**DOI:** 10.1111/eva.13249

**Published:** 2021-05-29

**Authors:** Lídia Escoda, Jose Castresana

**Affiliations:** ^1^ Institute of Evolutionary Biology (CSIC‐Universitat Pompeu Fabra) Barcelona Spain

**Keywords:** complete genome, *Galemys pyrenaicus*, heterozygosity, Iberian desman, Pyrenean desman, runs of homozygosity

## Abstract

The Pyrenean desman (*Galemys pyrenaicus*) is a small semiaquatic mammal endemic to the Iberian Peninsula. Despite its limited range, this species presents a strong genetic structure due to past isolation in glacial refugia and subsequent bottlenecks. Additionally, some populations are highly fragmented today as a consequence of river barriers, causing substantial levels of inbreeding. These features make the Pyrenean desman a unique model in which to study the genomic footprints of differentiation, bottlenecks and extreme isolation in an endangered species. To understand these processes, the complete genome of the Pyrenean desman was sequenced and assembled using a Bloom filter‐based approach. An analysis of the 1.83 Gb reference genome and the sequencing of five additional individuals from different evolutionary units allowed us to detect its main genomic characteristics. The population differentiation of the species was reflected in highly distinctive demographic trajectories. In addition, a severe population bottleneck during the postglacial recolonization of the eastern Pyrenees created one of the lowest genomic heterozygosity values recorded in a mammal. Moreover, isolation and inbreeding gave rise to a high proportion of runs of homozygosity (ROH). Despite these extremely low levels of genetic diversity, two key multigene families from an eco‐evolutionary perspective, the major histocompatibility complex and olfactory receptor genes, showed heterozygosity excess in the majority of individuals, revealing that functional diversity can be maintained up to a certain extent. Furthermore, these two classes of genes were significantly less abundant than expected within ROH. In conclusion, the genomic landscape of each analysed Pyrenean desman turned out to be strikingly distinctive and was a clear reflection of its recent ancestry and current conservation conditions. These results may help characterize the genomic health of each individual, and can be crucial for the conservation and management of the species.

## 1 INTRODUCTION

Complete genomes of endangered species are helping to identify features of individuals and populations that can be critical for in situ and ex situ conservation (Abascal et al., 2016; Benazzo et al., 2017; Ekblom et al., 2018; Humble et al., 2020; Saremi et al., 2019; Westbury et al., 2018; Xue et al., 2015; Zhu et al., 2018). To adequately manage species, it is essential to know not only which populations may be most threatened, but also which individuals from healthy populations may be optimal for genetic rescue or captive breeding. One of the important characteristics that must be considered in conservation is genetic diversity, which can be measured in individuals as the proportion of heterozygous positions in the genome. The heterozygosity rate or SNP density has been shown to vary greatly between different species (Prado‐Martinez et al., 2013). When considering only mammalian species of conservation concern, this value can be as low as 14 heterozygous sites or SNPs per million bases (SNPs/Mb) in a population of Channel Island fox (*Urocyon littoralis*) on a small oceanic island (Robinson et al., 2016, 2018) and can reach as many as 1,200 SNPs/Mb in some orangutan (*Pongo* sp.) populations (Locke et al., 2011), thus extending over two orders of magnitude. Low heterozygosity is generally caused either by population bottlenecks that have occurred in recent evolutionary history or current population declines, and it is unclear as to whether there is a critical heterozygosity value below which an individual or population can be considered at risk.

More important than the average heterozygosity rate is the variability of genetic diversity in the genome caused by different phenomena such as inbreeding. If inherited from a recent common ancestor, both copies of some chromosome blocks can be identical in inbred individuals, forming the so‐called runs of homozygosity (ROH) (Ceballos et al., 2018). The proportion of the genome in ROH is one of the different ways to determine the inbreeding coefficient. The ROH content not only informs about a fundamental characteristic of genomes, but can also provide a time frame of its origin, as recent inbreeding is expected to result in long ROH, while older inbreeding, for example due to past bottlenecks, is reflected in short ROH (Kardos et al., 2018). In many species, inbreeding leads to reduced fitness mainly due to the presence of detrimental mutations in homozygosis (Charlesworth & Willis, 2009). When inbreeding is widespread in a population and there is a positive correlation between individual inbreeding coefficients and fitness, inbreeding depression may occur, often leading to an extinction vortex in the short term (Kardos et al., 2016; Mills, 2013). Nevertheless, this pernicious association is not seen in populations in which lethal mutations have been purged during bottlenecks in their recent population history (Keller & Waller, 2002). In any case, knowing the inbreeding coefficient of individuals is critical for managing populations of conservation concern (Leroy et al., 2018; Supple & Shapiro, 2018).

Proteins that directly interact with the environment, such as those involved in the recognition of pathogens or the detection of chemical signals, are particularly interesting in the context of genetic diversity, as these require a high degree of inter‐ and intra‐locus variability to function properly. The major histocompatibility complex (MHC) is a large genomic region containing some of the key components of the immune system in vertebrates (Knapp, 2005; Vandiedonck & Knight, 2009). The main genes in this region are the MHC class I (MHC‐I) and MHC class II (MHC‐II) genes. Both types of genes encode surface proteins that bind to antigens derived from intracellular origin, in the case of MHC‐I, or extracellular, in the case of MHC‐II, and present them to different cells of the immune response (Neefjes et al., 2011). These are two multigene families of which there are several paralogues and pseudogenes. The gene content of the MHC region, both in the number of MHC duplicated genes and pseudogenes, is highly variable among mammals (Abduriyim et al., 2019; Papenfuss et al., 2015). Due to balancing selection, a huge diversity of alleles is found in all MHC genes, with tens to hundreds of alleles present in natural populations (Radwan et al., 2020; Sommer, 2005). This extraordinary genetic polymorphism ensures the recognition of a large diversity of antigens and reactivity against a wide spectrum of pathogens.

The olfactory receptor (OR) genes are the largest multigene family in mammals (Niimura & Nei, 2007). They are transmembrane proteins that function as receptors of various odour molecules in the environment. The genes are distributed on all chromosomes, forming genomic clusters (Glusman et al., 2001). OR loci have been shown to have more SNPs than expected due to the advantage conferred by the heterozygous state (Alonso et al., 2008). Interestingly, OR repertoires are highly distinctive in mammalian species with different niches such as terrestrial or aquatic (Hayden et al., 2010; Hughes et al., 2018), showing the ecological relevance of these genes.

Genes that are usually highly variable such as MHC and OR raise the interesting question of whether their genetic variability can be maintained when populations have extremely low genetic diversity or significant inbreeding levels (Aguilar et al., 2004; Marmesat et al., 2017). Another interesting question is whether ROH regions in highly inbred individuals can include genes for which heterozygosity is an advantage or whether these genes are less abundant in ROH (Kardos et al., 2018; Pemberton et al., 2012). The complete genomes of species with high inbreeding levels may help to address these questions.

The Pyrenean desman (*Galemys pyrenaicus*) is a small semiaquatic mammal belonging to the subfamily Desmaninae, a lineage that was composed of a large number of species during the Neogene (McKenna et al., 1997). However, a high extinction rate in this lineage led to only two species remaining in this subfamily today, making the Pyrenean desman an exceptional mammalian species from an evolutionary point of view. Its biological and ecological features are also remarkable. The Pyrenean desman is endemic to the north of the Iberian Peninsula, where it occupies small rivers and streams with well‐oxygenated and unpolluted waters. This habitat is only found today in mountain areas, and therefore, the distribution of the species is limited to some large mountain ranges of the Iberian Peninsula, which makes the distribution of the species very restricted and patchy. The species has conspicuous adaptations to the aquatic environment, such as its characteristic snout and its webbed limbs, which it uses to capture the benthic invertebrates on which it feeds underwater (Kryštufek & Motokawa, 2018; Palmeirim & Hoffmann, 1983). Due to its shrinking distribution, the Pyrenean desman is classified as vulnerable in the IUCN Red List and some of its populations are highly threatened (Fernandes et al., 2008). One of the biggest problems to study this species derives from its mostly nocturnal habits and the fact that it can rarely be observed, making it difficult to get a clear picture of basic aspects of its biology, starting with its distribution. Surveys of the species have been greatly facilitated in recent years thanks to the location of the excrements that the desman deposits on river rocks, and the genetic determination of these excrements (Igea et al., 2013), which led to the recent discovery of new populations. Phylogeographic studies showed that the genetic structure of the species is very strong, being subdivided into five populations (evolutionarily significant units) that probably arose as a consequence of isolation in different glacial refugia (Igea et al., 2013; Querejeta et al., 2016). A ddRAD‐based study on the species revealed extremely low heterozygosity in some individuals from the eastern Pyrenees (Querejeta et al., 2016), probably as a consequence of repeated bottlenecks during the postglacial recolonization of these mountains (Gillet et al., 2017). In addition, kinship networks revealed that there are important connectivity problems for the species in some places, since individuals from nearby rivers show little relatedness. Both ecological and artificial barriers affect connectivity of the Iberian desman populations, but specially large dams have been shown to cause total isolation of populations in the upper parts of rivers, leading to extremely high inbreeding levels in the individuals confined to these areas (Escoda et al., 2017, 2019).

The exceptional evolutionary history of the Pyrenean desman and the current state of extreme isolation of some of its populations make this small mammal a unique species into which study fundamental biological and ecological questions. At the same time, this research can contribute to the conservation of this endangered species. Despite its interest, no genome from this species has been obtained so far. Having the complete genome of the Pyrenean desman would allow us to answer interesting questions raised in previous work and address completely new ones. In particular, we can study how the combination of strong population bottlenecks and high inbreeding levels is reflected in the genomic landscape of a threatened species like this one. We can also try to understand how a species with such extremely low genetic diversity can survive and what are the features of genes that usually have high genetic variability, such as MHC and OR genes. Here, we provide the first draft genome assembly and annotation of the Pyrenean desman and resequence five additional individuals with the objective of addressing these questions and the ultimate goal of obtaining useful information that can be applied to the conservation and management of endangered species.

## 2 MATERIALS AND METHODS

### 2.1 Genome sequencing and assembly

We selected six Pyrenean desmans for genome sequencing. Two of them (IBE‐C3734 and IBE‐C3773) had been utilized in a previous study (Escoda et al., 2017), and we extracted DNA from the rest of the samples using the methods described in that report (Table S1). All the samples used in this study were minimally invasive samples obtained as part of works with the species promoted by environmental authorities or came from animals found dead during these surveys. The DNA quality of the samples selected for genome sequencing was controlled by checking for the absence of smearing in a gel electrophoresis.

A single male specimen (IBE‐C5619) was used for sequencing the reference genome. Its genomic DNA was shotgun‐sequenced using three Illumina TruSeq DNA PCR‐free libraries, two with an insert size of 350 bp and one of 550 bp, and two mate‐pair libraries with insert sizes of 5 and 9 kb, respectively. For the five additional resequenced genomes, a TruSeq DNA PCR‐free library with an insert size of 350 bp was constructed for each individual. All the libraries were prepared by Macrogen Inc. (South Korea).

We separated the different sequences of the mate‐pair libraries with NxTrim v0.4.3 (O'Connell et al., 2015), and for the assembly, we used only the fraction with mate‐pair orientation and complete reads, that is, with no adapter sequence (called ‘unknown’ in NxTrim), which produced the best results in initial assemblies. Then, we used fastp v0.19.5 (Chen et al., 2018) with all the libraries to remove adapters and low complexity reads (to eliminate sequencing artefacts), as well as reads with a quality score of lower than 20 or a length of less than 150 bp. Using the same tool, the reads were base‐corrected.

To predict the genome size of the Pyrenean desman, we first used Jellyfish v2.2.10 (Marçais & Kingsford, 2011) to determine the frequency distribution of 21 mers in filtered Illumina sequencing data from the largest library (C5619_60 Gb, Table S2). With the distribution obtained, we used GenomeScope 2.0 (Ranallo‐Benavidez et al., 2020) to estimate the genome size.

Using the filtered reads, we assembled the de novo genome using a strategy with relatively low computational memory requirements based on the use of the Bloom filter option implemented in ABySS v2.1.5 (Jackman et al., 2017). Since this method has not been thoroughly tested for large genomes, we first searched for the optimal parameters of both contigs and scaffolds formation stages: *k*‐mer size (*k*), minimum *k*‐mer count threshold for Bloom filter assembly (kc), minimum number of pairs required for building contigs (*n*) and minimum number of pairs required for building scaffolds (*N*). All the assemblies were carried out with the following parameters in common, as preliminary analyses showed them to be the best: Bloom filter size (*B*) of 80G, number of Bloom filter hash functions (*H*) of 4 and minimum untig size required for building contigs (*s*) of 1,000.

We used QUAST v5.0.2 (Gurevich et al., 2013) to compute the summary statistics and BUSCO (Benchmarking Universal Single‐Copy Orthologs) v3.0.2 (Simão et al., 2015) with the mammalia_odb9 database to assess the genome completeness of the different assemblies. The best assembly parameters were chosen to maximize the N50 of the assembly and the number of core genes found with BUSCO, as well as to minimize the number of scaffolds and gaps (Ns). The GC content was calculated using BEDTools (Quinlan & Hall, 2010) in 100‐kb windows.

### 2.2 Gene prediction

We identified the repetitive regions in the genome assembly with RepeatMasker v4.0.7 (http://www.repeatmasker.org) using the Dfam Consensus release 20170127 and Repbase release 20170127 databases (Jurka et al., 2005). Complex repeats were hard‐masked, whereas simple repeats were soft‐masked so that they could be used in some further steps.

Due to the endangered status of the Pyrenean desman, obtaining fresh tissues of sufficient quality to perform RNA‐Seq analysis for gene prediction was not possible. Therefore, we used a homology‐based approach in which we optimized parameters and protein databases to be included in order to achieve the largest possible number of predicted genes. We also checked the alignments with mammalian homologues of specific control proteins such as the titin, the longest protein in mammals (Labeit & Kolmerer, 1995), and the MHC‐I and MHC‐II genes, the most variable genes in the mammalian genome. For gene detection, we used MAKER2 v2.31.10 (Holt & Yandell, 2011) with the masked genome sequence (with contigs ≥ 1,000 bp). Gene prediction and training of the different prediction methods involved were performed through two iterative rounds using a pipeline previously described (Fitak et al., 2016). In the first round, genes were predicted using two methods: directly from protein homology (option protein2genome = 1) using exonerate (Slater & Birney, 2005), and also with AUGUSTUS v3.3.2 (Stanke et al., 2006), previously trained with a small fraction of the genome. For the protein homology prediction, we used the proteomes from four species of the Eulipotyphla order to which the Pyrenean desman belongs: *Condylura cristata*, *Sorex araneus*, and *Erinaceus europaeus*, all of them unpublished genomes from the Broad Institute available at GenBank (Clark et al., 2016), and *Solenodon paradoxus* (Casewell et al., 2019). In addition, we included the human proteome available at GenBank, as its completeness allowed us to detect additional genes. These proteomes were also used as protein evidence, as well as to refine the gene models using exonerate. In the second round, genes were predicted using AUGUSTUS, as before, and also with SNAP version 2013‐02‐16 (Korf, 2004), the latter trained with predictions of the first MAKER2 round. For the two rounds, an expected maximum intron size of 15,000 bp was used and scaffolds were divided into chunks of 400,000 bp. Gene annotation of the generated GFF and FASTA files was based on a BLAST search (Altschul et al., 1997) against the mammal section of the UniProt/Swiss‐Prot database (UniProt Consortium, 2019). GenomeTools v1.5.10 (Gremme et al., 2013) was used to compute statistics on the predicted genes.

MHC‐I proteins are composed of two subunits, α and β, of which only the first is encoded by a gene in the MHC region. The α chain of MHC‐I proteins can be encoded by genes with a variable number of exons ranging from 5 to 9 (Papenfuss et al., 2015), most typically 8. Accordingly, MHC‐I genes were retrieved using the terms ‘class I histocompatibility antigen’ and ‘alpha chain’ from the GFF and FASTA files. Out of 45 genes found, 26 with between 5 and 8 exons were retained for analysis. Genes with a lower or higher exon number had more problematic alignments with other mammalian homologues and, therefore, could be pseudogenes. For the heterozygous sequences, we selected the assembled sequence. Alignments at the amino acid level of these sequences together with those from other mammals (Abduriyim et al., 2019), including *C. cristata* and a selection of human genes, were generated with MAFFT v7.464 (Katoh & Standley, 2013) and processed with Gblocks v0.91 (Castresana, 2000) to remove poorly aligned positions using low stringency conditions (minimum length of a block of 5 and allowing positions with gaps in half the number of sequences). Then, a maximum‐likelihood phylogenetic tree was reconstructed with RAxML v8.2.12, using a JTT model of amino acid substitution and a gamma distribution of evolutionary rates (Stamatakis, 2014).

To identify olfactory receptor (OR) genes, we used 659 putative OR genes obtained from the first round of MAKER2 annotated as ‘olfactory receptor’ in the GFF file, as this round contained a much larger number of putative OR genes obtained by protein homology than the second one. We classified this set of putative OR genes into functional genes and nonfunctional pseudogenes with the olfactory receptor family assigner (ORA) BioPerl module (Hayden et al., 2010). In addition, four genes with large numbers of masked sequences and a gene that was very divergent in initial trees were removed. The alignment and phylogenetic tree of the final 529 genes together with those from *C. cristata* and human were generated as described above.

### 2.3 Read mapping and variant calling

Cleaned reads of each individual were mapped to the de novo reference genome using BWA v0.7.17 (Li & Durbin, 2009). Subsequently, SAMtools v1.9 (Li et al., 2009) was used to produce BAM alignments of scaffold length greater than 1,000 bp in which duplicated reads were removed and only unambiguously mapped and properly paired reads with a minimum mapping quality (−*q*) of 30 were kept. Variant calling was carried out with BCFtools v1.9 (Li, 2011) from the BAM alignments in scaffolds with a minimum length of 40,000 bp. This threshold was selected because we observed that, according to the result of Qualimap v2.2.2 (Okonechnikov et al., 2016), the mapping quality and insert size graphs greatly improved in scaffolds longer than this length, including their two ends (where mapping quality was reduced in smaller scaffolds), something essential for proper genetic diversity estimates. Additional filtering parameters for obtaining the final VCF files of the genotypes included a minimum variant quality (−*Q*) of 30, a maximum depth of coverage of twice the mean genome‐wide coverage of each individual (as determined with Qualimap) and a minimum depth of coverage of 10, which was chosen after testing different values of this parameter.

### 2.4 Genomic heterozygosity

To detect sex‐linked scaffolds, we computed the mean coverage of scaffolds longer than 40,000 bp in all individuals with SAMtools. We based the classification of the scaffolds into chromosome classes on the fact that the ratio of coverage between the female and any of the males presented three clearly delimited groups: autosomes (ratio ~1), X chromosomes (~2) and Y chromosomes (~0). We considered those with a ratio of coverage of between 0 and 0.04 to be Y chromosome scaffolds, and those with a ratio between 1.5 and 2.5 to be X chromosome scaffolds. After excluding Y and X chromosome scaffolds identified in this way, 583 putative autosomal scaffolds longer than 40,000 bp, totalling 1.722 Gb, remained. These autosomal scaffolds were the basis of further analyses.

Genome‐wide heterozygosity estimation was based on the heterozygous genotypes of each individual obtained after discarding homozygous sites that were either identical to the reference genome or alternative homozygous sites for that individual, therefore keeping only heterozygous sites. The number of heterozygous sites was obtained with the ‐SNPdensity option of VCFtools v0.1.16 (Danecek et al., 2011) from the VCF files of each individual (filtered as described above) using different values of the minimum depth of coverage ranging from 4 to 20. To estimate the heterozygosity rate, the number of heterozygous sites was divided by the total number of called sites passing the same quality filters and that had the same minimum depth of coverage in the corresponding BAM files, as calculated with SAMtools. That is, both the numerator and the denominator passed the same filters to obtain the heterozygosity rate.

To examine the effect of the genome coverage on the heterozygosity analyses, we also calculated the genome‐wide heterozygosity of two downsampled genomes corresponding to the two individuals with the highest genome coverage (IBE‐C5619 and IBE‐BC2778). For this purpose, we used a single 13× library for the individual IBE‐C5619 (C5619_20 Gb, Table S2) and a 15× subsample of the library for the individual IBE‐BC2778 obtained with seqtk (available at https://github.com/lh3/seqtk).

The number of heterozygous positions in exons of different genes was calculated with BEDTools (Quinlan & Hall, 2010) by intersecting the VCF files of each individual with the BED files containing the exon positions of the desired genes present in the 583 autosomal scaffolds longer than 40,000 bp. To estimate the heterozygosity rate of each gene, the resulting number of heterozygous positions was divided by the total number of exon positions that passed the quality filters (as described above) and had a minimum depth of coverage of 10 for that individual. This was calculated for 22 MHC‐I genes and 507 OR genes as well as for the set of all predicted genes located in autosomal scaffolds longer than 40,000 bp, for which heterozygous positions were determined.

### 2.5 Runs of homozygosity

We identified ROH using four different approaches. Firstly, we used BCFtools/RoH (Narasimhan et al., 2016), which applies a hidden Markov model, with default parameters except that the window size was 100 kb. Secondly, we used PLINK v1.90p (Purcell et al., 2007) to detect ROH segments larger than 100 kb with default options, in which a scanning window of 50 sites can contain, at most, 1 heterozygous position. For these two methods, we used as input the merged VCF file of all the individuals. For the final calculation of the proportion of ROH in the genome, we only considered the fraction of the genome in autosomal scaffolds longer than 100 kb (1.715 Gb in 467 scaffolds).

We also used two additional methods that are based on each individual genome for identifying ROH. We first used ROHan (Renaud et al., 2019), which simultaneously estimates heterozygosity and identifies ROH regions from the BAM file of each individual genome, using 100‐kb windows and allowing 1 heterozygous position per 100 kb (the default value). Finally, we used a simple method based on the SNP count in 100‐kb windows. For this purpose, we calculated the number of SNPs of the autosomal scaffolds with VCFtools v0.1.16 (Danecek et al., 2011), in 100‐kb nonoverlapping windows, using the VCF files of each individual. Then, we calculated the proportion of 100‐kb windows that contained 0 heterozygous sites, which were considered ROH windows, with respect to all the 100‐kb windows (not including partial windows at scaffold ends). For this estimation, allowing no heterozygous sites in the windows gave the most similar average to the inbreeding coefficients calculated from ddRAD data for two of the desmans (Escoda et al., 2017). In order to examine the effect of the genome coverage on the ROH analyses, we also performed the same calculations with the downsampled genomes corresponding to individuals IBE‐C5619 and IBE‐BC2778. We also used the ROH identified with BCFtools/RoH to construct plots and distributions of the cumulative genome fraction in ROH of different lengths.

The proportion of exons of MHC‐I and OR in ROH was calculated with BEDTools (Quinlan & Hall, 2010) by intersecting the BED files containing the exon positions of the desired genes with the BED files of ROH and non‐ROH 100‐kb windows of each individual (as determined from the simple SNP count in 100‐kb windows). The proportion was calculated as the number of exons in ROH windows divided by the total number of exons in 100‐kb windows. To calculate the p‐value, the ROH and non‐ROH 100‐kb windows of each individual were randomized 1,000 times while maintaining the proportion of windows with 0 heterozygous positions corresponding to each individual. This was calculated for 19 MHC‐I genes and 477 OR genes as well as for the set of all predicted genes located in autosomal scaffolds longer than 100,000 bp, for which ROH regions were determined.

### 2.6 Genetic structure and demographic history

To assemble the mitochondrial genomes, we mapped the raw reads of each individual to a published complete mitochondrial genome (Cabria et al., 2006) using BWA v0.7.17 (Li & Durbin, 2009) and called variants using BCFtools v1.9 (Li, 2011). We obtained each mitochondrial genome by applying the variants of each individual to the reference mitogenome with the BCFtools consensus tool. All the mitogenomes were aligned using MAFFT (Katoh & Standley, 2013), and a maximum‐likelihood phylogenetic tree was reconstructed with RAxML using a GTR model of nucleotide substitution and a gamma distribution of evolutionary rates (Stamatakis, 2014).

The principal component analysis (PCA) was performed with the KING toolset (Manichaikul et al., 2010).

For the pairwise sequentially Markovian coalescent (PSMC) analysis (Li & Durbin, 2011), we used the genotypes to generate a consensus FASTA sequence of the autosomal genome scaffolds. We performed the PSMC analyses using the following parameters, as suggested in the program manual (https://github.com/lh3/psmc): maximum number of iterations (*N*) of 25, maximum coalescent time (*t*) of 15, initial theta/rho ratio (*r*) of 5 and parameter pattern (*p*) of ‘4+25*2+4+6’. The above parameters were able to provide good resolution and showed more than 10 recombination events in each of the atomic time intervals within 20 iterations. Similar results were found when using the alternative parameters: *N* = 25, *t* = 5, *r* = 1 and *p *= ‘4+30*2+4+6+10’ (Nadachowska‐Brzyska et al., 2016). We assessed the variance of the analyses using 100 bootstrap replicates of each individual. The final estimates of population size and time were scaled with a mutation rate of 5 × 10^−9^ mutations/site/generation and a generation time of 2 years. The Pyrenean desman can live up to 4 years, and occasionally as long as 6 (Gonzalez‐Esteban et al., 2002), while reconstructed pedigrees (Escoda et al., 2019) suggest that 2 years approximates well to the average intergeneration interval. The mutation rate per generation of 5 × 10^−9^ was selected to be similar to other species with short generation times (Smeds et al., 2016; Uchimura et al., 2015), as expected (Piganeau & Eyre‐Walker, 2009); the resulting per year mutation rate for the desman (2.5 × 10^−9^ mutations/site/year) was similar to the mammalian average of 2.2 × 10^−9^ mutations/site/year (Kumar & Subramanian, 2002). To examine the effect of the genome coverage on the PSMC analyses, we performed the same analysis with the two downsampled genomes of the individuals IBE‐C5619 and IBE‐BC2778.

## 3 RESULTS

### 3.1 Pyrenean desman samples sequenced

**FIGURE 1:**
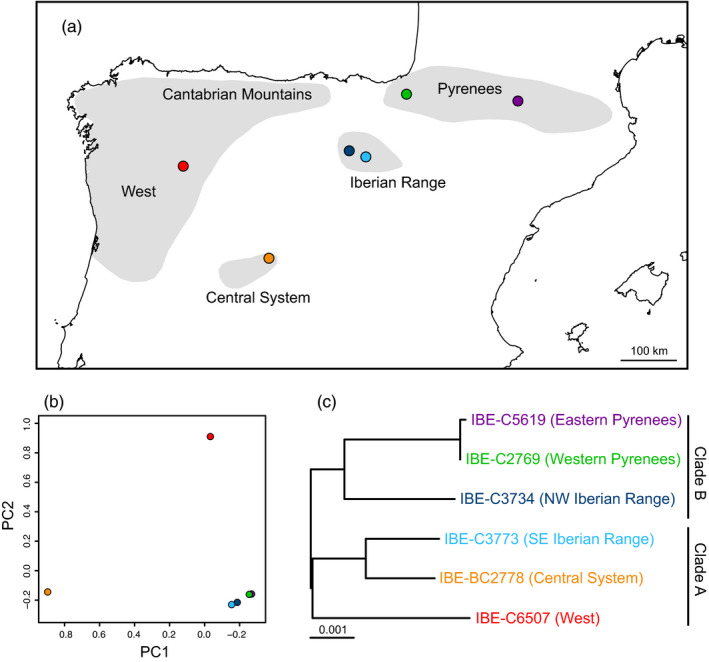
(a) Map of the Iberian Peninsula showing the distribution of the Pyrenean desman (shadowed areas) and the locations of the sequenced individuals. (b) Principal component analysis of the genotypes. (c) Maximum‐likelihood phylogenetic tree of the mitochondrial genomes, in which the main clades are indicated. The root of the tree was placed between clades A and B, and the scale is in substitutions/site

A total of six Pyrenean desman individuals covering the majority of the species distribution range (Figure 1, Table S1) were sequenced. The reference genome was sequenced from a male from the eastern Pyrenees at 121× coverage (Table S2), while five additional desmans from other locations (western Pyrenees, northwest and southeast Iberian Range, Central System, and West of the Iberian Peninsula, i.e., four out the five main populations) were resequenced with coverages ranging from 10.3× to 33.3× (Figure 1, Table S3).

### 3.2 Genome assembly and Bloom filter parameterization

A total of 32 combinations of Bloom filter parameters, *k*‐mer values and other ABySS options were tested for the desman genome assembly (Table S4). The parameters that gave the best overall results were a minimum *k*‐mer count threshold for the Bloom filter (kc) of 2, a Bloom filter size (*B*) of 80G and a number of Bloom filter hash functions (*H*) of 4, together with a *k*‐mer size (*k*) of 100 and other ABySS parameters detailed in Table S4. We obtained a final assembly of 1.83 Gb, slightly larger than the genome size of 1.74 Gb estimated by GenomeScope. The final assembly had a scaffold N50 of 8.5 Mb (Table S5) and 96.3% of the mammalian BUSCO core genes (Table S6). Although assemblies with higher N50 values were obtained, these involved a larger number of scaffolds, more gaps or lower numbers of BUSCO genes (Table S4). We further evaluated the accuracy of the desman genome assembly by mapping the short‐insert sequencing data to the assembled genome: 99.9% of the reads were mapped and a low‐coverage peak corresponding to the Y and X chromosomes was observed, as expected for a male (Figure S1A). The GC content was 41.7% and showed a variability across chromosomal regions typical of a mammalian genome (Figure S2).

### 3.3 Gene prediction

After detecting repetitive elements (Table S7) and masking the genome, we predicted 20,936 protein‐coding genes using the MAKER2 pipeline (Holt & Yandell, 2011). The annotation edit distance (AED) of the genes, which provides a measure of the prediction congruence, showed that 95% of the genes have a score lower than 0.5 (Figure S1B), indicating a well‐annotated genome (Campbell et al., 2014). Other features of the predicted genes that indicated that the Bloom filter‐based assembly of the Pyrenean desman is equivalent to any other properly assembled mammalian genome include a bimodal distribution of intron length (Figure S1C), as observed in other mammalian genomes (Piovesan et al., 2016), and some genes with coding sequences (CDS) longer than 100,000 bp (Figure S1D), such as titin (Labeit & Kolmerer, 1995).

The gene sequences of two important multigene families were retrieved and aligned. The phylogenetic trees of 26 MHC‐I (Figure S3A) and 529 OR (Figure S3B) genes together with those of other mammals indicated a large diversity of genes in both families. The number of OR genes found in the Pyrenean desman is typical of terrestrial and semiaquatic species and not of aquatic mammals, in which many genes have been lost (Hayden et al., 2010; Hughes et al., 2018), as expected.

### 3.4 Genomic heterozygosity

We estimated genome‐wide heterozygosity using different values of the minimum depth of coverage, which ranged from 4 to 20 (Figure S4A). We found no relevant differences in the estimated heterozygosity rates of all individuals for minimum depth values between 4 and 14, but heterozygosity decreased substantially at minimum depths of 16 or higher for the two individuals sequenced with the lowest coverage (IBE‐C2769 and IBE‐C3734, respectively), thus preventing the use of minimum depths ≥16 for all individuals. For the downsampled libraries of individuals IBE‐C5619 and IBE‐BC2778, heterozygosity rates were lower than for the original libraries, although by a very small amount for IBE‐C5619. For the downsampled genome of IBE‐BC2778, heterozygosity was underestimated around ~30% at most depths and only at a depth of 20 it was close to the original genome. It should also be taken into account that the number of sites passing the filters for the calculations decreased drastically at high minimum depths, especially those ≥12, for four individuals as a consequence of their lower genome coverage (Figure S4B). As a large number of sites was important for some analyses, we selected a minimum depth of coverage of 10 to be used in subsequent analyses. This value provided enough resolution for characterizing the heterozygosity rate while maintaining a sufficient number of genomic positions necessary for other analyses in all sequenced Pyrenean desmans. It also assumes that heterozygosity is underestimated for the genomes with the lowest coverage (IBE‐C2769, IBE‐C3734, IBE‐C3773 and IBE‐C6507), although by a magnitude that might not be very different from fluctuations found within populations. For the two genomes with the highest coverages (IBE‐C5619 and IBE‐BC2778), this depth value gives the same accuracy in the heterozygosity rate estimation than any other minimum depth (Figure S4A).

The autosomal genome heterozygosity rate calculated with a minimum depth of coverage of 10 varied greatly between the six desmans and is among the lowest found in mammals (Table S8; Figure S5). It ranged between 12 and 459 SNPs/Mb, with an average of 198 for all individuals. The underestimation of heterozygosity likely to occur in the four genomes with the lowest coverage cannot explain the large differences found between the desmans of different populations. The desman from the eastern Pyrenees, with 12 SNPs/Mb, has the lowest heterozygosity recorded in a mammal according to the published values so far (Figure S5), and due to the high coverage with which this genome was sequenced, it is unlikely to be underestimated.

### 3.5 Runs of homozygosity and inbreeding

**FIGURE 2:**
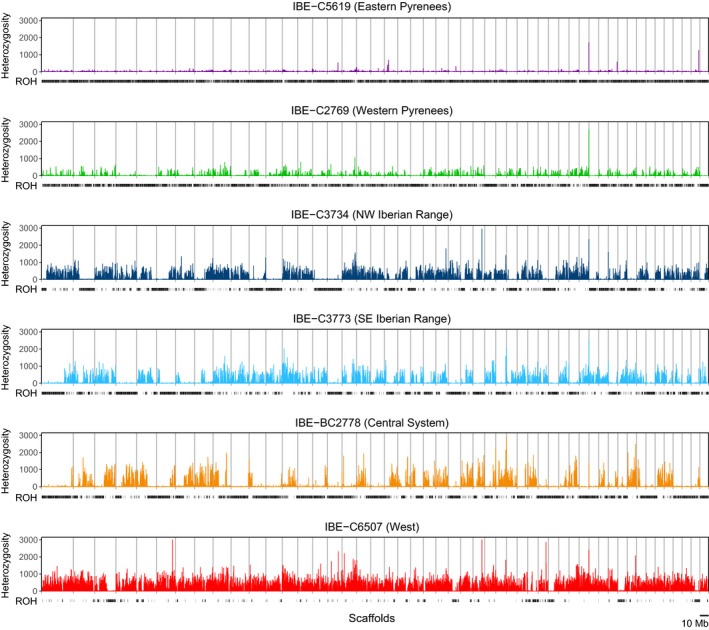
Heterozygosity rate, in SNPs/Mb, of the scaffolds longer than 10 Mb measured in 100‐kb windows for each Pyrenean desman genome. Two outlier windows with >3,000 SNPs/Mb (for individual IBE‐C6507) were truncated for visual purposes. The black lines under each graph indicate ROH regions (identified as 100‐kb windows with 0 SNPs). Scaffolds are ordered by size

When we calculated the heterozygosity in 100‐kb windows and plotted the values across the scaffolds, we found that most of the desmans presented very long ROH, with important variations in lengths and patterns among individuals (Figure 2). To calculate the proportion of ROH for the genome of each individual, which can be used to estimate the inbreeding coefficient, we applied four different approaches: PLINK, BCFtools/RoH, ROHan and the proportion of 100‐kb windows with 0 heterozygous positions. The values calculated with the four methods were highly correlated (Table S9), with all pairwise correlation coefficients being higher than 0.95 (Table S10), that is, the different methods behaved comparatively similar between individuals. However, the averages showed substantial differences among methods with the specific parameter settings used, with PLINK giving the maximum average (0.65) and the proportion of homozygous 100‐kb windows giving the minimum (0.45). These results indicate that methods for estimating ROH independently of the population background such as ROHan or the proportion of homozygous 100‐kb windows may have an adequate comparative value when no population data are available. They also show that all the desmans had very high values of the inbreeding coefficient. For example, when calculated as the proportion of homozygous 100‐kb windows, which gives the lowest values, they varied between 0.11 for the individual from the West of the Iberian Peninsula and 0.70 for the individual from the eastern Pyrenees (Table S9; Figure 2). For the downsampled genomes, all estimates of inbreeding levels were higher than those calculated with the equivalent high coverage genomes, likely due to lack of SNPs in ROH (Table S9). The magnitude of the variation found with the reduced and original genomes is not very different from that observed with the various estimation methods of the inbreeding coefficient based on ROH, but it should be noted that, for more precise estimates, high coverage genomes are necessary.

The plot of the cumulative genome fraction contained in ROH of different lengths (Figure S6A) showed that, except for the individual from the West population (IBE‐C6507), all desmans had a large proportion of its genome in long ROH (>1 Mb), therefore indicating abundant inbreeding events occurred in the last few generations. Specifically, all desmans except IBE‐C6507 had a proportion of their genome contained in long ROH greater than 0.38, with the individual IBE‐C5619 reaching 0.82 (Figure S6B).

### 3.6 Heterozygosity excess and ROH deficiency in MHC and OR genes

**FIGURE 3:**
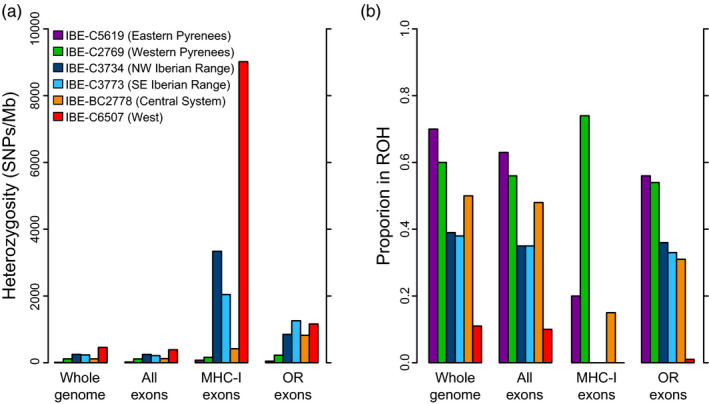
(a) Heterozygosity rate in exons of MHC‐I and OR genes of the sequenced Pyrenean desmans, given in SNPs/Mb, in comparison with the heterozygosity of the whole genome and all the exons. (b) Proportion in ROH regions of exons of MHC‐I and OR genes in comparison with the proportion of ROH calculated for the whole genome and all the exons (colours are as in panel a)

The low genomic heterozygosity observed in the whole genome is also reflected in the exons (mean of 185 SNPs/Mb across all exons and individuals; Table S11 and Figure 3A). A low genetic diversity may affect the adequate functioning of highly polymorphic genes, such as the MHC or OR genes. To understand how this extreme reduction in genetic variability affected these particular genes, we calculated the heterozygosity in their exons (Table S11 and Figure 3A). For the MHC‐I exons, most individuals presented much higher heterozygosity values (2,509 SNPs/Mb, i.e., 12.7 times higher on average than the whole genome). The desman from the West of the Iberian Peninsula showed the highest heterozygosity (9,018 SNPs/Mb, i.e., 19.6× excess), whereas the desmans from the western Pyrenees and the Central System showed a very low excess. OR exons also presented heterozygosity excess with respect to all exons (728 SNPs/Mb on average, representing a 3.7× excess). In this case, the heterozygosity excess was more similar between all the individuals. The underestimation of heterozygosity that may occur in the low‐coverage genomes should not affect these results assuming that coverage is equally distributed throughout the genome, although the results with these individuals should be treated more carefully.

Another important question is whether these highly polymorphic genes are contained or not within ROH segments. If the presence of such genes in ROH is suboptimal, a lower proportion of their exons is expected in ROH. The expected value is the proportion of the genome in ROH, in other words, an estimate of the inbreeding coefficient, for example calculated as the proportion of homozygous 100‐kb windows (Table S9). When calculated for the entire set of exons, the proportion of exons in ROH was smaller than expected (0.41 vs. 0.45 on average; Table S12 and Figure 3B). Despite this small difference, it was highly significant for all the individuals (*p* < 0.001), so it seems that there is certain ROH deficiency in coding regions. The situation was most striking for MHC‐I, as the proportion of exons in ROH was much lower than expected (0.18), and highly significant for five of the individuals, with *p*‐values of between <0.001 and 0.01. There was also a significantly smaller proportion of OR exons in ROH (0.35), although in this case only three of the individuals showed a significantly lower proportion of OR exons in ROH (*p* < 0.001).

The high heterozygosity observed in MHC‐I genes could be attributed to assembly problems in the MHC region. If this were the case for any specific gene, mapping of paralogues could cause a higher depth of coverage in these regions compared with the depth of the whole genome and a high heterozygosity for all individuals in that gene. To test these two ideas, we first calculated the depth of coverage in MHC‐I exons and observed that they had a similar depth of coverage to the set of all exons and the whole genome for all individuals (Table S13). Then, we determined the number of SNPs in each of the MHC‐I genes for all individuals and observed that this number varied widely between individuals (Table S14). Most importantly, for all genes, some individuals had 0 SNPs, meaning that they were homozygous for that gene, and even in some genes, all individuals were homozygous. That is, not all individuals were highly heterozygous for a specific gene, which would have been an indication of assembly problems in those genes, but rather only some individuals were homozygous. We can then infer that the high diversity observed in some individuals is due to the presence of different alleles, as expected for MHC evolution. This result and the normal depth of coverage found in these genes are indirect indications of no relevant assembly artefacts behind the high heterozygosity detected in some MHC‐I genes of certain individuals. Similar results of heterozygosity and depth of coverage were observed for OR genes (Tables S14 and S15), suggesting that they were also properly assembled.

### 3.7 Genetic structure and demographic history

The PCA of the genotypes agreed, in general terms, with the geographic proximity of the individuals (Figure 1B). On the other hand, the maximum‐likelihood phylogenetic tree of the assembled mitochondrial genomes (Figure 1C) showed the two main mitochondrial clades of the species and an important mito‐nuclear discordance for the individual from the SE Iberian Range, whose geographic proximity and nuclear similarity to the other individual from the Iberian Range is not reflected in the mitochondrial tree, corroborating previous work (Escoda et al., 2017; Igea et al., 2013; Querejeta et al., 2016).

**FIGURE 4:**
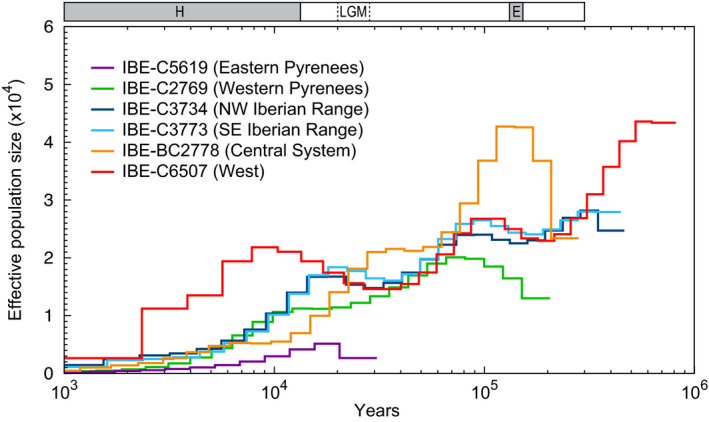
Historical effective population size of the Pyrenean desman individuals inferred by PSMC. The result is scaled with a mutation rate of 5 × 10^−9^ mutations/site/generation and an average generation time of 2 years. The last two interglacial periods, Holocene (H) and Eemian (E), are indicated with grey boxes and the last glacial maximum (LGM) with dashed lines

The PSMC analysis revealed that all the populations experienced a general decline together with substantial fluctuations in their effective sizes during the time covered by the plot, of which the last ~300 thousand years showed the best resolution (Figures 4 and S7). When compared with the major climatic events that occurred in this time interval (Clark et al., 2009; Dahl‐Jensen et al., 2013), the two population size peaks observed are close to the beginning of the two interglacial periods of this time (Eemian and Holocene). Within this general pattern, there were important differences among individuals. The demographic fluctuation patterns were similar for the two desmans from the Iberian Range and, to a certain extent, the one from the western Pyrenees. The individual from the West of the Iberian Peninsula showed a delayed decline and the highest current effective population size. The desman from the Central System presented a high population size peak during the Eemian interglacial and a large decline since then. Finally, the curve of the desman from the eastern Pyrenees revealed an extremely small effective population size and its data only covered a short period of time, probably due to its exceptionally low heterozygosity. To test whether differences in coverage affected these results, we performed the PSMC analysis with downsampled genomes from the two individuals with the highest coverage (IBE‐C5619 and IBE‐BC2778). Similar results were found for the downsampled and original genomes in both individuals (Figure S8), indicating that the coverage of these genomes does not significantly alter the PSMC results.

## 4 DISCUSSION

### 4.1 Bloom filter assembly of a mammalian genome

Important progress has been made in genome sequencing technologies in recent years, leading to a decreased cost per base and a huge increase in the number of short sequences retrieved, and allowing the de novo assembly of multiple species genomes with great coverage and quality (Goodwin et al., 2016). Nevertheless, the enormous quantity of data generated by these platforms has created new computational problems in terms of assembling large genomes, as this requires vast computational resources, especially memory (Sohn & Nam, 2018). Of the algorithms that reduce overall memory requirements, Minia (Chikhi & Rizk, 2013) and ABySS 2.0 (Jackman et al., 2017) use a Bloom filter to represent the de Bruijn graph, making it possible to assemble large genomes on low‐memory computers. However, this Bloom filter‐based approach has only been used in a few cases so far (Arnason et al., 2018; Renaut et al., 2018), probably because the method depends on a number of parameters that are not yet well understood and need to be tested. Here, we show that the Bloom filter available in ABySS can be used to assemble the genome of the Pyrenean desman and produce a high‐quality draft genome, with a scaffold N50 of 8.5 Mb and 96.3% of the BUSCO core genes. The final assembly was carried out in just 10 hours, using a computer with 128 GB of RAM memory and 16 processors. Part of the efficiency when assembling the Pyrenean desman genome could be related to the extremely low heterozygosity of the individual selected for the de novo assembly, which is one of the most important factors in obtaining a good assembly (Bradnam et al., 2013). It is clear, however, that newer sequencing technologies based on long reads enable better assemblies, even with chromosome‐scale scaffolds (Sohn & Nam, 2018). However, the reasonable computation time necessary to assemble a large genome with a Bloom filter and the possibility to run the program on a local computer has a number of advantages. For example, it allowed us to test many different settings, not only to properly adjust the Bloom filter parameters, but also to apply other conditions to obtain the best possible assembly. It may also facilitate work in remote settings with limited Internet access such as field stations. Most importantly, the moderate computational expense associated with this assembly methodology can help promote the sequencing of reference genomes in more species of conservation concern (McMahon et al., 2014).

### 4.2 Population demographic history of a species with low dispersal capacity

One of the most important life‐history characteristics of the Pyrenean desman to help us understand the peculiar genomic features revealed in this work is its low dispersal capacity. The desman is morphologically well adapted to the aquatic medium, but its terrestrial locomotion is slow and laboured (Palmeirim & Hoffmann, 1983), meaning that, with a few exceptions, its dispersal occurs only via the river network. Among the most important consequences of this low dispersal potential was the generation of a strong genetic structure during glacial periods, probably due to the complete isolation of glacial refugia over long periods, giving rise to five highly differentiated populations and strict contact zones with very low rates of mixing between adjacent populations (Escoda et al., 2017; Igea et al., 2013; Querejeta et al., 2016). During the period covered by the PSMC plot, there were important fluctuations in the size of these Pyrenean desman populations (Figure 4). Within a general trend of population decrease, two size peaks are apparent. Interestingly, they are close to the beginning of the two main interglacial periods of the last ~300 thousand years (Eemian and Holocene). An expansion of the fluvial network during the deglaciations could have increased the extent of the favourable habitat for the Pyrenean desman and, consequently, its population size. In addition, substantial differences between the demographic trajectories of the specimens sequenced were observed, much greater than the differences that are typically found between individuals of the same population (Nadachowska‐Brzyska et al., 2016). These contrasting demographic histories are consistent with the different conditions likely to have been experienced by the Pyrenean desman populations during the glacial periods, and support the treatment of these populations as distinct evolutionarily significant units (Igea et al., 2013; Querejeta et al., 2016), which should be managed independently in conservation programmes (Coates et al., 2018; Funk et al., 2012).

Range expansions and recurring bottlenecks in evolutionary history or recent past of a population can lead to a significant reduction in its genetic diversity (Excoffier et al., 2009; Hewitt, 2000) and, consequently, the individual heterozygosity rate. The sequencing of species of conservation concern has led to comparisons being made between the heterozygosity rates of different species (Figure S5). Very rarely does a mammal have less than 100 SNPs/Mb; prior to this study, the lowest heterozygosity rate had been found in a Channel Island fox, with 14 SNPs/Mb, on the island of San Nicolas (Robinson et al., 2016). The Pyrenean desmans sequenced in this work span a wide range of heterozygosity rates, in line with the highly different evolutionary histories of the desman populations, with values running from 459 SNPs/Mb in the specimen from the West of the Iberian Peninsula to just 12 in the desman from the eastern Pyrenees (Table S8). The latter is now the lowest heterozygosity rate recorded in any mammal, to our knowledge. This extremely low value suggests that the number of founding members of the population, situated at the eastern edge of the species range, could be as low as the number of foxes that colonized the small oceanic island of San Nicolas. The desmans from the western Pyrenees and the Central System are also positioned towards the bottom of the heterozygosity rate list (Figure S5), highlighting the ecological and evolutionary interest of these populations.

### 4.3 Lessons from the genome of a species with extraordinary inbreeding levels

The reduced overland dispersal capacity of the Pyrenean desman has had profound effects on this species, not only during its recent history, but also in the present. Due to the abundance of artificial and ecological barriers in many of the rivers inhabited by this species, connectivity through the river network is currently greatly diminished. Large hydroelectric dams and water reservoirs very effectively block the movement of the desman. Additionally, the concatenation of smaller artificial barriers and ecological barriers resulting from contamination and predation by invasive species in the lower parts of rivers has confined many desman populations to the river headwaters over the past few generations (Quaglietta et al., 2018). The consequence of this isolation is that desmans can only breed with other individuals of the same river, which are usually closely related as determined through relatedness networks (Escoda et al., 2017, 2019). This, in turn, leads most desmans to have high inbreeding levels (Escoda et al., 2017). Considering that the inbreeding coefficient for the offspring of two first‐degree relatives is 0.25 (Weir et al., 2006), values higher than this can only be achieved through continuous mating between closely related individuals for several generations. Five of the desmans sequenced in this study presented inbreeding coefficients greater than 0.25 (Table S9; proportion of homozygous 100‐kb windows). In fact, part of the ROH, the shortest runs, is due to more ancient population bottlenecks, whereas the longest runs are due to recent inbreeding (Ceballos et al., 2018; Kardos et al., 2018). The proportion of short ROH (<1 Mb) was substantial for the different individuals (Figure S6B), indicating that all populations have been affected by a significant reduction in population size in the past, and not only those with the lowest heterozygosity values such as those from the Pyrenees and the Central System. For most individuals, however, recent inbreeding appears to be the major contributor to their ROH content, as demonstrated by the large proportion of long ROH in them (Figure S6B).

The genomic sequences of individuals of an endangered species like the Pyrenean desman can also help determine the functional genomic features of particular specimens, to gain a better understanding of their genomic health (Díez‐del‐Molino et al., 2018; Steiner et al., 2013). In this work, we have characterized two groups of proteins from multigenic families in which high levels of diversity are essential, both at the inter‐ and intra‐locus levels: the class I major histocompatibility complex and the olfactory receptors. The analysis of the genetic diversity in these revealed interesting differences between the sequenced individuals. This was particularly true of the MHC‐I genes, which must maintain high levels of genetic diversity to cope with external pathogens (Radwan et al., 2020). For example, the desman from the West of the Iberian Peninsula and, to a certain extent, the two specimens from the Iberian System maintain levels of heterozygosity in the MHC‐I genes that are much higher than in other parts of the genome, while this effect was much smaller in the other sequenced desmans. In principle, balancing selection could be acting in some populations to compensate for the sharp decrease occurred in heterozygosity throughout the genome due to the bottlenecks (Aguilar et al., 2004; Marmesat et al., 2017). However, we found that MHC‐I genes tend to be absent from ROH regions, so this mechanism could also be important for maintaining genetic diversity where it is most necessary in highly inbred populations. A similar ROH deficiency in the MHC regions was found in the genome of cattle breeds (Zhang et al., 2015). The OR genes also presented a consistent heterozygosity excess in most desmans, and the numbers of these genes were similarly reduced in ROH, particularly in the desman from the West of the Iberian Peninsula, in which almost no OR gene is present in ROH. Therefore, an evolutionary mechanism through which MHC‐I and OR genes are negatively selected in ROH regions may be acting. Since both MHC‐I and OR genes are clustered in the genome, just a few regions could be targeted by this type of selection: individuals without ROH in them would have higher fitness and chances of surviving. However, a population genomic analysis with more individuals per population and with high genome coverage to obtain more accurate estimates is necessary to corroborate these results and thoroughly understand how highly inbred specimens and populations cope with the need to maintain certain levels of genetic diversity in these important genes.

A fundamental question that remains to be answered regarding the Pyrenean desman is whether these populations can survive with extremely low genome‐wide heterozygosity (particularly in the eastern Pyrenees, where the sequenced desman had 12 SNPs/Mb), high proportion of ROH, and precariously maintained functional genetic diversity. Despite the shrinking habitat and range of this species (Fernandes et al., 2008), desmans are currently surviving with these poor genomic health indicators in the small river stretches to which the populations have become constrained. There is apparently no signal of generalized reduced fitness that may point to inbreeding depression, and new juveniles are detected every year, although we still do not know if some of these populations or all will collapse in the future. The reason why they continue to survive today may lie in a possibly low load of deleterious alleles in the population. The bottlenecks experienced by the Pyrenean desman during the glaciations, as well as other adverse climatic periods such as droughts, could have purged deleterious and lethal mutations from the genomic background of the species, meaning that homozygosis is not as problematic today in the desman as it is in other species that present higher long‐term genetic diversity but also more lethal equivalents (Keller & Waller, 2002; Leberg & Firmin, 2008). A similar situation of a species surviving with an extremely low heterozygosity (14 SNPs/Mb) has been reported for the island fox of the San Nicolas population (Robinson et al., 2018). Although not with such low values, there are several other species with heterozygosity values that range between 100 and 120 SNPs/Mb (Figure S5; including the Iberian desmans from the western Pyrenees and Central System), in which it is likely that a similar mechanism of purging of lethal mutations occurred (Morin et al., 2020; Westbury et al., 2018). Further analysis of nonsynonymous substitutions and functional variation in populations of endangered species will be necessary to test these hypotheses.

Even if they survive, such low‐diversity and highly inbred desman populations could be extremely vulnerable to the effects of pandemics caused by new pathogens, which may affect all individuals of the population similarly (De Castro & Bolker, 2004; Pedersen et al., 2007). Consequently, careful protection and monitoring of these populations are necessary. If population reinforcement becomes necessary in order to reduce inbreeding, it should involve specimens from the same evolutionary unit and be planned with great caution because these genetically low‐diversity populations might be particularly difficult to rescue, as there are high chances of introducing elevated levels of recessive mutations from large populations (Kyriazis et al., 2021; Robinson et al., 2018). For this reason, any conservation strategies should preferentially promote natural connectivity between nearby river populations or, where this is not feasible, proceed with reciprocal translocations between recently disconnected populations. Genomics can help to not only determine which specimens may be more or less appropriate for genetic rescue or captive breeding according to different genomic health indicators of each individual, particularly inbreeding (Leroy et al., 2018; Supple & Shapiro, 2018), but also monitor future individuals sampled after the conservation actions to confirm whether the measures employed are helping to improve the impoverished genomic health of the Pyrenean desman.

## CONFLICTS OF INTEREST

None declared.

## Supporting information

Supplementary Material

## Data Availability

Sequence data and the genome are available under NCBI BioProject PRJNA705855. Additionally, the genotype files and key commands used in the analyses are available in Dryad (https://doi.org/10.5061/dryad.3r2280gd9).
