# Supplementary material for: The genome of the Pyrenean desman and the effects of bottlenecks and inbreeding on the genomic landscape of an endangered species
Source: Evol Appl. 2021 May 29;14(7):1898–913. doi: 10.1111/eva.13249 (PMC8288019; doi:10.1111/eva.13249)
Supplement: Supplementary file 1 — Supplementary Material [file EVA-14-1898-s001.pdf]

Supporting Information: Tables and Figures

The genome of the Pyrenean desman and the effects of bottlenecks and inbreeding on the genomic landscape of an endangered species

Lídia Escoda [1], Jose Castresana [1]

[1] Institute of Evolutionary Biology (CSIC-Universitat Pompeu Fabra), Passeig Marítim de la Barceloneta 37, 08003 Barcelona, Spain

Corresponding author: Jose Castresana
Email: jose.castresana@csic.es

Index of Supporting Information

Table S1. Specimens used in this study, sample types, sex, sampling year, locality, and geographical area.
Table S2. Summary statistics of the *de novo* genome sequencing data.
Table S3. Summary statistics of the genome sequencing data of the additional individuals.
Table S4. Summary statistics of the genome assemblies using different combinations of parameters in ABySS.
Table S5. Summary statistics of the genome assembly.
Table S6. Summary of the BUSCO analysis.
Table S7. Summary statistics of the repetitive elements.
Table S8. Autosomal genome-wide heterozygosity of the sequenced Pyrenean desmans.
Table S9. Runs of homozygosity (ROH) of the sequenced Pyrenean desmans.
Table S10. Pearson's correlation coefficients between the different estimates of ROH.
Table S11. Heterozygosity values in exons of the MHC-I and olfactory receptor genes of the sequenced Pyrenean desmans.
Table S12. Proportion in ROH regions of exons of the MHC-I and olfactory receptor genes of the sequenced Pyrenean desmans.
Table S13. Depth of coverage of exons of the MHC-I and olfactory receptor genes in comparison with the average depth of coverage for the whole genome.
Table S14. Number of SNPs found for each MHC-I gene.
Table S15. Number of SNPs found for each olfactory receptor gene.
Figure S1. Distributions showing the main features of the Bloom filter-based genome assembly of the Pyrenean desman and the predicted protein-coding genes.
Figure S2. GC content variation in the autosomal scaffolds longer than 10 Mb.
Figure S3. Phylogenetic trees of the MHC-I and olfactory receptor genes.
Figure S4. Genome-wide heterozygosity rate for different values of minimum depth of coverage.
Figure S5. Genome-wide heterozygosity rate for different mammalian species of conservation concern.
Figure S6. Cumulative proportion of the genome contained in ROH of different lengths.
Figure S7. Historical effective population size inferred from the Pyrenean desman genomes by PSMC with 100 bootstrap replicates.
Figure S8. Historical effective population size inferred from downsampled genomes.

Table S1. Specimens and sample types used in this study together with information about their sex, sampling year, locality, geographical area, and autonomous commnunity. Two specimens used in a previous study are indicated.

Specimen code	Sample type	Sex	Year	Locality	Geographical area
IBE-C5619	Tissue	Male	2017	Torán	Eastern Pyrenees (Catalunya)
IBE-C2769	Tail tip	Male	1999	Ezpelura-Urrotz	Western Pyrenees (Navarra)
IBE-C3734 (1)	Tail tip	Female	2011	Oja	Northwestern Iberian Range (La Rioja)
IBE-C3773 (1)	Tail tip	Male	2011	Mayor	Southeastern Iberian Range (La Rioja)
IBE-BC2778	Tissue	Male	2019	Hija de Dios	Central System (Castilla y León)
IBE-C6507	Tissue	Male	2018	Requejo	West of the Iberian Peninsula (Castilla y León)

[1] Escoda L, González-Esteban J, Gómez A, Castresana J (2017) Using relatedness networks to infer contemporary dispersal: application to the endangered mammal *Galemys pyrenaicus*. *Molecular Ecology*, **26**, 3343–3357.

Table S2. Summary statistics of the *de novo* genome sequencing data.

Library name	Library insert size (bp)	Read length (bp)	Raw reads	Filtered reads	Filtered bases	Coverage	Illumina platform	NCBI SRA accession number
C5619_20Gb	350	150	168,103,284	158,251,220	23,737,683,000	13.0	HiSeq X Ten	SRR13862646
C5619_50Gb	350	150	353,994,004	333,733,964	50,060,094,600	27.4	HiSeq X Ten	SRR13862645
C5619_60Gb	550	150	663,416,860	625,115,962	93,767,394,300	51.3	NovaSeq 6000	SRR13862644
C5619_5k	5,000	150	69,131,334	69,034,668	10,313,274,039	5.6	NovaSeq 6000	SRR13862643
C5619_9k	9,000	150	313,352,690	290,403,706	43,426,665,509	23.8	NovaSeq 6000	SRR13862642
**Total**			**1,567,998,172**	**1,476,539,520**	**221,305,111,448**	**121.0**		

Table S3. Summary statistics of genome sequencing data of the additional individuals.

Library name	Library insert size (bp)	Read length (bp)	Raw reads	Filtered reads	Filtered bases	Coverage	Illumina platform	NCBI SRA accession number
C2769_20Gb	350	150	133,311,010	125,595,550	18,839,332,500	10.3	HiSeq X Ten	SRR13862641
C3734_20Gb	350	150	164,266,428	156,862,252	23,529,337,800	12.9	HiSeq X Ten	SRR13862640
C3773_20Gb	350	150	180,798,280	171,444,472	25,716,670,800	14.1	HiSeq X Ten	SRR13862639
BC2778_50Gb	350	150	423,518,938	406,106,310	60,915,946,500	33.3	NovaSeq 6000	SRR13862638
C6507_20Gb	350	150	186,336,338	175,033,874	26,255,081,100	14.4	HiSeq X Ten	SRR13862637

Table S4. Summary statistics of the genome assemblies using different combinations of parameters in ABySS. All the assemblies have the following parameters in common: Bloom filter size (B) = 80G, number of Bloom filter hash functions (H) = 4, and minimum untig size required for building contigs (s) = 1000. The final assembly chosen is shown in bold.

	**Abyss parameters**				**Contigs**					**Scaffolds**					**BUSCO analysis**				
**#**	**k**	**kc**	**n**	**N**	**Number**	**N50**	**Total length (bp)**	**Largest sequence (bp)**	**N's**	**Number**	**N50**	**Total length (bp)**	**Largest sequence (bp)**	**N's**	**Comp.**	**Sing.**	**Dup.**	**Frag.**	**Miss.**
1	80	2	5	5	111,433	32,553	1,785,457,123	291,897	332,861	26,042	1,200,095	1,830,800,637	9,588,943	47,235,273	3,899	3,877	22	133	72
2	80	2	5	10	111,433	32,553	1,785,457,123	291,897	332,861	23,940	5,951,183	1,832,660,941	23,566,475	49,089,317	3,940	3,919	21	103	61
3	80	2	10	5	113,947	31,323	1,785,225,099	291,897	397,393	25,437	1,269,001	1,830,952,436	12,846,275	47,722,165	3,904	3,885	19	127	73
4	80	2	10	10	113,947	31,323	1,785,225,099	291,897	397,393	23,437	5,697,658	1,832,922,782	23,203,185	49,721,941	3,944	3,924	20	97	63
5	80	3	5	5	109,933	33,225	1,785,420,032	385,381	400,687	25,857	1,213,311	1,830,181,266	8,465,635	46,605,776	3,903	3,880	23	128	73
6	80	3	5	10	109,933	33,225	1,785,420,032	385,381	400,687	23,766	6,073,268	1,832,267,957	28,195,572	48,689,944	3,941	3,919	22	99	64
7	80	3	10	5	112,616	31,855	1,785,480,117	347,451	460,921	25,328	1,249,154	1,830,590,970	9,531,084	47,091,628	3,906	3,886	20	129	69
8	80	3	10	10	112,616	31,855	1,785,480,117	347,451	460,921	23,302	5,554,286	1,832,778,480	28,196,456	49,317,935	3,947	3,928	19	94	63
9	90	2	5	5	82,588	45,555	1,796,039,717	443,520	349,179	16,098	1,791,614	1,827,110,659	17,686,848	32,988,668	3,934	3,917	17	100	70
10	90	2	5	10	82,588	45,555	1,796,039,717	443,520	349,179	14,975	7,181,995	1,829,147,288	23,672,106	35,023,894	3,952	3,931	21	85	67
11	90	2	10	5	85,338	43,273	1,796,092,999	443,520	417,713	15,683	1,807,790	1,827,675,698	9,450,852	33,699,235	3,929	3,907	22	106	69
12	90	2	10	10	85,338	43,273	1,796,092,999	443,520	417,713	14,622	7,459,917	1,829,836,990	36,183,241	35,866,583	3,947	3,925	22	90	67
13	90	3	5	5	82,394	46,348	1,795,401,409	410,797	458,625	16,825	1,722,795	1,826,834,836	9,026,015	33,376,920	3,918	3,899	19	111	75
14	90	3	5	10	82,394	46,348	1,795,401,409	410,797	458,625	17,247	7,154,225	1,828,635,784	34,866,347	35,215,360	3,953	3,932	21	88	63
15	90	3	10	5	85,015	44,011	1,795,415,076	473,012	529,804	18,377	1,755,848	1,827,249,887	11,938,933	33,967,457	3,919	3,902	17	116	69
16	90	3	10	10	85,015	44,011	1,795,415,076	473,012	529,804	15,002	7,263,463	1,829,516,963	36,159,321	36,235,826	3,948	3,930	18	89	67
17	100	2	5	5	63,049	63,603	1,803,404,661	480,138	442,655	13,929	2,363,627	1,826,436,552	17,794,570	25,161,174	3,936	3,914	22	97	71
18	100	2	5	10	63,049	63,603	1,803,404,661	480,138	442,655	12,831	8,511,288	1,828,107,683	34,981,249	26,855,549	3,947	3,922	25	92	65
19	100	2	10	5	64,927	60,334	1,803,400,543	463,860	501,639	14,683	2,457,278	1,826,595,599	12,564,118	25,472,454	3,940	3,916	24	97	67
**20**	**100**	**2**	**10**	**10**	**64,927**	**60,334**	**1,803,400,543**	**463,860**	**501,639**	**12,306**	**8,503,682**	**1,828,347,170**	**36,404,611**	**27,224,426**	**3,953**	**3,931**	**22**	**86**	**65**
21	100	3	5	5	69,357	63,700	1,800,943,157	726,128	648,716	18,162	2,158,393	1,828,462,988	12,351,970	29,828,976	3,936	3,914	22	97	71
22	100	3	5	10	69,357	63,700	1,800,943,157	726,128	648,716	17,270	7,464,961	1,830,000,858	34,975,378	31,398,726	3,946	3,923	23	90	68
23	100	3	10	5	70,076	60,387	1,801,259,151	713,669	747,718	16,607	2,285,473	1,828,365,895	10,356,824	29,475,535	3,934	3,913	21	103	67
24	100	3	10	10	70,076	60,387	1,801,259,151	713,669	747,718	15,878	7,378,031	1,830,046,073	35,735,109	31,189,660	3,948	3,925	23	93	63
25	110	2	5	5	64,540	76,058	1,806,041,319	686,611	650,423	20,494	2,922,818	1,830,260,978	14,191,065	26,895,880	3,938	3,912	26	102	64
26	110	2	5	10	64,540	76,058	1,806,041,319	686,611	650,423	18,701	9,154,820	1,832,069,487	31,306,718	28,726,537	3,950	3,925	25	90	64
27	110	2	10	5	63,849	73,186	1,806,339,970	686,611	742,276	17,004	3,310,031	1,830,799,709	17,730,278	27,184,354	3,937	3,913	24	100	67
28	110	2	10	10	63,849	73,186	1,806,339,970	686,611	742,276	16,524	9,577,427	1,831,694,757	31,308,620	28,141,420	3,956	3,934	22	82	66
29	110	3	5	5	99,452	70,759	1,793,541,866	710,746	1,036,511	47,086	2,620,137	1,838,978,192	11,612,014	48,562,092	3,892	3,868	24	133	79
30	110	3	5	10	99,452	70,759	1,793,541,866	710,746	1,036,511	48,094	9,109,270	1,835,154,356	36,456,462	44,722,154	3,900	3,876	24	117	87
31	110	3	10	5	95,217	68,549	1,794,502,044	686,728	1,196,453	41,311	2,648,512	1,838,754,245	13,241,708	47,426,515	3,892	3,871	21	128	84
32	110	3	10	10	95,217	68,549	1,794,502,044	686,728	1,196,453	42,202	8,942,909	1,836,068,300	26,818,792	44,770,810	3,926	3,904	22	95	83

k: size of k-mer; kc: minimum k-mer count threshold for Bloom filter assembly; n: minimum number of pairs required for building contigs; N: minimum number of pairs required for building scaffolds; Comp.: Complete BUSCOs; Sing.: Complete single-copy BUSCOs; Dup.: Complete duplicated BUSCOs; Frag.: Fragmented BUSCOs, Miss.: Missing BUSCOs.

Table S5. Summary statistics of the genome assembly. All the statistics are based on contigs of size >= 500 bp.

**Statistics**	**Contigs**	**Scaffolds**
Sequence count	64,927	12,306
Total length (bp)	1,803,400,543	1,828,347,170
Largest contig (bp)	463,860	36,404,611
N's	501,639	27,224,426
N50 (count)	60,334 (8,720)	8,503,682 (66)
N75 (count)	31,301 (19,089)	3,815,754 (140)

Table S6. Summary of the BUSCO analysis using 4,104 mammalian single-copy orthologs database.

Statistics	Count	Ratio (%)
Complete BUSCOs	3,953	96.3
Complete single-copy BUSCOs	3,931	95.8
Complete duplicated BUSCOs	22	0.5
Fragmented BUSCOs	86	2.1
Missing BUSCOs	65	1.6

Table S7. Summary statistics of the repetitive elements identified with RepeatMasker.

TE class	Count	Length (bp)	Percentage of sequence (%)
SINEs:	746,460	150,640,913	8.24
Alu/B1	28	788	0.00
MIRs	266,561	35,470,149	1.94
LINEs:	548,054	204,676,726	11.19
LINE1	361,334	161,962,297	8.86
LINE2	157,413	37,140,022	2.03
L3/CR1	23,967	4,506,547	0.25
RTE	4,583	968,770	0.05
LTR elements:	264,663	80,383,117	4.40
ERVL	51,842	18,704,034	1.02
ERVL-MaLRs	82,131	24,678,381	1.35
ERV_classI	87,954	29,637,393	1.62
ERV_classII	24,854	2,370,471	0.13
DNA elements:	199,476	38,364,850	2.10
hAT-Charlie	108,626	19,788,936	1.08
TcMar-Tigger	41,077	9,333,173	0.51
Unclassified:	3,288	570,873	0.03
Small RNA:	20,790	1,797,159	0.10
Satellites:	55,522	19,797,132	1.08
Simple repeats:	405,554	18,609,900	1.02
Low complexity:	84,860	4,446,792	0.24
**Total interspersed repeats**		**474,636,479**	**25.96**

Table S8. Autosomal genome-wide heterozygosity of the sequenced Pyrenean desmans, given in SNPs/Mb. The total positions column corresponds to the called sites for each individual passing quality filters and with a minimum depth of coverage of 10. The average for the six individuals is also given. Specimen codes indicated with an asterisk (*) correspond to downsampled genomes.

Specimen code	Heterozygous positions	Total positions	Genomic heterozygosity
IBE-C5619	19,800	1,696,316,846	12
IBE-C2769	67,844	585,552,814	116
IBE-C3734	255,322	1,015,658,628	251
IBE-C3773	276,081	1,178,708,185	234
IBE-BC2778	195,591	1,685,426,942	116
IBE-C6507	572,825	1,248,283,120	459
IBE-C5619*	9,202	1,101,716,283	8
IBE-BC2778*	116,376	1,422,639,220	82
**Average**			198

Table S9. Runs of homozygosity (ROH) of the sequenced Pyrenean desmans estimated with different methods. Averages for the six individuals are also given. Specimen codes indicated with an asterisk (*) correspond to downsampled genomes.

Specimen code	PLINK	BCFtools/ RoH	ROHan	Proportion of homozygous 100-kb windows
IBE-C5619	0.97	0.83	0.72	0.70
IBE-C2769	0.83	0.64	0.69	0.60
IBE-C3734	0.57	0.38	0.44	0.39
IBE-C3773	0.59	0.38	0.45	0.38
IBE-BC2778	0.74	0.51	0.63	0.50
IBE-C6507	0.19	0.10	0.13	0.11
IBE-C5619*	0.99	0.92	0.84	0.83
IBE-BC2778*	0.82	0.68	0.68	0.66
**Average**	**0.65**	**0.47**	**0.51**	**0.45**

Table S10. Pearson's correlation coefficients between the different estimates of runs of homozygosity (ROH) of the sequenced Pyrenean desmans.

	PLINK	BCFtools /RoH	ROHan	Proportion of homozy-gous 100-kb windows
**PLINK**	-			
**BCFtools/RoH**	0.98	-		
**ROHan**	0.99	0.97	-	
**Proportion of homozygous 100-kb windows**	0.98	0.99	0.98	-

Table S11. Heterozygosity values in exons of the MHC-I and olfactory receptor genes of the sequenced Pyrenean desmans, given in SNPs/Mb, in comparison with the expected heterozygosity (extracted from Table S8). The number of total exon positions analyzed for each protein class and individual is given in brackets (the analysis is based on autosomal scaffolds > 40,000 bp and positions passing quality filters with a minimum depth of coverage of 10). Averages across all individuals are also given.

Specimen code	Expected heterozygosity	All exons	MHC-I	Olfactory receptor
IBE-C5619	12	22 (33,389,605)	76 (26,400)	48 (456,877)
IBE-C2769	116	114 (12,816,399)	161 (6,194)	225 (124,366)
IBE-C3734	251	248 (21,603,728)	3,338 (15,578)	851 (255,128)
IBE-C3773	234	212 (25,027,950)	2,043 (18,109)	1,258 (272,590)
IBE-BC2778	116	125 (33,189,561)	419 (26,247)	821 (440,964)
IBE-C6507	459	390 (25,006,310)	9,018 (19,074)	1,162 (310,580)
**Average**	**198**	**185**	**2,509**	**728**

Table S12. Proportion in ROH regions of exons of the MHC-I and olfactory receptor genes of the sequenced Pyrenean desmans in comparison with the expected proportion (proportion of homozygous 100-kb windows taken from Table S9). The p-value is given in brackets. Average across individuals as well as the total number of exons analyzed for each protein class are also given (the analysis is based on autosomal scaffolds > 100,000 bp).

**Specimen code**	**Expected proportion**	**All exons**	**MHC-I**	**Olfactory receptor**
IBE-C5619	0.70	0.63 (0.00)	0.20 (0.00)	0.56 (0.00)
IBE-C2769	0.60	0.56 (0.00)	0.74 (0.79)	0.54 (0.11)
IBE-C3734	0.39	0.35 (0.00)	0.00 (0.00)	0.36 (0.29)
IBE-C3773	0.38	0.35 (0.00)	0.00 (0.00)	0.33 (0.16)
IBE-BC2778	0.50	0.48 (0.00)	0.15 (0.01)	0.31 (0.00)
IBE-C6507	0.11	0.10 (0.00)	0.00 (0.00)	0.01 (0.00)
**Average**	**0.45**	**0.41**	**0.18**	**0.35**
Exons analyzed		173,056	115	477

Table S13. Depth of coverage of exons of MHC-I and olfactory receptor genes in comparison with the average depth of coverage for the whole genome (estimated with QualiMap for scaffolds > 1,000 bp) for all individuals.

Specimen code	Whole genome	All exons	MHC-I	Olfactory receptor
IBE-C5619	75.4	76.0	70.4	73.7
IBE-C2769	8.1	8.7	7.6	7.5
IBE-C3734	10.3	11.0	10.5	9.8
IBE-C3773	11.0	12.3	11.5	10.2
IBE-BC2778	27.7	28.4	27.4	27.4
IBE-C6507	11.4	12.0	11.9	11.1

Table S14. Number of SNPs found in each MHC-I gene from Table S11 for each individual. Red boxes indicate genes with 0 SNPs for that individual.

Gene	IBE-C5619	IBE-C2769	IBE-C3734	IBE-C3773	IBE-BC2778	IBE-C6507
GAL0_00004542	0	0	0	1	0	0
GAL0_00004541	0	1	1	1	0	0
GAL0_00004686	2	0	3	0	0	2
GAL0_00004354	0	0	0	1	0	1
GAL0_00004353	0	0	0	0	0	19
GAL0_00004392	0	0	1	1	0	28
GAL0_00004393	0	0	0	0	0	21
GAL0_00004391	0	0	6	6	11	66
GAL0_00004389	0	0	13	10	0	19
GAL0_00004215	0	0	1	0	0	0
GAL0_00004224	0	0	5	4	0	3
GAL0_00004218	0	0	1	1	0	0
GAL0_00004223	0	0	1	1	0	3
GAL0_00004220	0	0	0	1	0	0
GAL0_00004217	0	0	0	0	0	0
GAL0_00004216	0	0	2	2	0	0
GAL0_00004219	0	0	0	0	0	0
GAL3_00002566	0	0	0	0	0	0
GAL3_00005138	0	0	0	0	0	5
GAL0_00004358	0	0	2	2	0	0
GAL0_00004226	0	0	15	3	0	1
GAL0_00004225	0	0	1	3	0	4

Table S15. Number of SNPs found in each olfactory receptor gene from Table S11 for each individual. Red boxes indicate genes with 0 SNPs for that individual.

Gene	IBE-C5619	IBE-C2769	IBE-C3734	IBE-C3773	IBE-BC2778	IBE-C6507
OR-001	0	0	0	0	0	0
OR-002	0	0	0	0	0	0
OR-003	0	0	0	0	0	2
OR-004	0	0	0	0	0	0
OR-005	0	0	0	0	1	0
OR-006	0	0	0	0	1	0
OR-007	0	0	2	2	3	0
OR-008	0	0	1	0	1	2
OR-009	0	0	7	0	3	0
OR-010	0	0	1	0	0	1
OR-011	0	0	0	0	0	0
OR-012	0	0	0	0	0	0
OR-013	0	0	1	0	1	0
OR-014	0	0	0	0	0	0
OR-015	0	0	0	0	0	0
OR-016	0	0	0	0	0	0
OR-017	0	0	0	0	2	0
OR-018	0	0	0	0	0	0
OR-019	0	0	0	0	0	0
OR-020	0	0	0	0	0	0
OR-021	0	0	2	0	1	1
OR-022	0	0	0	0	0	1
OR-023	0	0	0	0	0	2
OR-024	0	0	0	0	0	3
OR-025	0	0	0	0	0	1
OR-026	0	0	0	0	0	0
OR-027	0	0	0	0	0	0
OR-028	0	0	1	0	0	2
OR-029	0	0	0	0	1	0
OR-030	0	0	0	0	1	1
OR-031	0	0	7	0	14	1
OR-032	0	0	0	0	0	2
OR-033	0	0	0	0	0	3
OR-034	8	3	7	5	6	3
OR-035	0	0	0	0	0	2
OR-036	0	0	0	1	0	0
OR-037	0	0	22	45	61	0
OR-038	0	0	0	1	0	0
OR-039	0	0	0	1	0	0
OR-040	0	0	0	0	0	0
OR-041	0	0	0	0	0	0
OR-042	0	0	0	0	1	0
OR-043	0	0	3	0	0	0
OR-044	0	0	0	1	0	0
OR-045	0	0	0	0	0	0
OR-046	0	0	27	12	6	9
OR-047	0	0	0	0	0	0
OR-048	0	0	0	0	0	0
OR-049	0	0	0	1	0	0
OR-054	0	0	0	0	0	0
OR-055	0	0	0	0	0	0
OR-056	0	0	0	0	0	0
OR-057	0	0	0	0	0	0
OR-058	0	0	0	0	0	0
OR-059	3	0	0	1	3	11
OR-060	0	0	0	1	0	0
OR-061	0	5	10	4	0	0
OR-062	0	0	0	0	0	0
OR-063	0	0	0	1	0	1
OR-064	0	0	0	7	0	4
OR-065	0	0	0	0	0	0
OR-066	0	0	0	1	0	0
OR-067	0	0	0	0	0	2
OR-068	0	0	0	0	0	1
OR-069	0	0	0	0	0	0
OR-070	0	0	0	0	0	0
OR-071	0	0	0	0	0	2
OR-072	0	0	0	0	0	0
OR-073	0	0	0	3	0	2
OR-074	0	0	0	0	0	0
OR-075	0	0	0	0	0	8
OR-076	0	0	0	0	0	8
OR-077	0	0	0	0	0	0
OR-078	0	0	0	0	0	0
OR-079	0	0	0	1	0	1
OR-080	0	0	0	0	0	0
OR-081	0	0	0	0	0	0
OR-082	0	0	0	1	0	1
OR-083	0	0	0	1	0	1
OR-084	0	0	0	0	0	0
OR-085	0	0	0	0	0	0
OR-086	0	0	0	0	0	1
OR-087	0	0	0	0	0	0
OR-088	6	0	4	0	5	9
OR-089	0	0	0	0	0	0
OR-090	0	0	0	0	0	0
OR-091	0	0	0	0	0	0
OR-092	0	0	0	0	0	0
OR-093	0	0	0	0	0	0
OR-094	0	0	0	0	0	0
OR-095	0	0	0	0	0	0
OR-096	0	0	0	1	0	0
OR-097	0	3	0	17	0	3
OR-098	0	0	0	0	0	0
OR-099	0	0	0	0	0	1
OR-100	0	0	0	0	0	0
OR-101	0	0	0	0	0	0
OR-102	0	0	2	0	0	0
OR-103	0	0	1	0	0	0
OR-104	0	0	0	0	0	1
OR-105	0	0	0	0	0	1
OR-106	0	0	0	0	0	0
OR-107	0	0	0	0	1	1
OR-108	0	0	0	0	1	0
OR-109	0	0	1	0	1	1
OR-110	0	0	0	0	1	1
OR-111	0	0	0	0	2	4
OR-112	0	0	0	0	0	0
OR-113	0	0	0	0	0	0
OR-115	0	0	0	0	5	1
OR-116	0	0	0	1	0	0
OR-118	0	0	0	4	0	5
OR-119	0	0	0	1	1	0
OR-120	0	0	0	0	1	0
OR-121	0	0	0	0	1	0
OR-122	0	0	0	3	0	0
OR-123	0	0	0	0	1	0
OR-124	0	0	0	0	3	1
OR-125	0	0	0	0	0	0
OR-127	0	0	0	0	0	1
OR-128	0	0	0	3	0	1
OR-129	0	0	0	0	0	0
OR-132	0	0	2	0	0	1
OR-133	0	0	0	0	0	0
OR-134	0	0	0	0	0	0
OR-135	0	0	0	0	0	0
OR-136	0	0	0	0	0	0
OR-137	0	0	0	0	0	0
OR-138	0	0	0	0	0	0
OR-139	0	0	1	0	0	0
OR-140	0	0	0	0	0	0
OR-141	0	0	0	0	0	0
OR-142	0	0	0	0	0	0
OR-143	0	0	0	0	0	0
OR-144	0	0	0	0	0	0
OR-145	0	0	0	0	0	0
OR-146	0	0	0	0	0	2
OR-147	0	0	0	0	0	0
OR-148	0	0	0	0	0	4
OR-149	0	0	0	0	0	0
OR-150	0	0	0	0	0	1
OR-151	0	0	0	0	0	1
OR-152	0	0	0	0	0	2
OR-153	0	0	0	0	0	1
OR-154	0	0	0	0	0	6
OR-155	0	0	0	0	0	0
OR-156	0	0	0	0	0	0
OR-157	0	0	0	0	0	2
OR-158	0	0	0	0	0	2
OR-159	0	0	0	0	0	0
OR-160	0	0	0	0	0	0
OR-161	0	0	0	0	0	0
OR-162	0	0	0	0	0	0
OR-163	0	0	0	0	0	0
OR-164	0	0	0	23	0	13
OR-165	0	0	0	0	0	0
OR-166	0	0	0	0	0	1
OR-167	0	0	0	0	0	0
OR-168	0	0	0	0	0	0
OR-169	0	0	0	0	0	0
OR-170	0	0	0	0	0	0
OR-171	0	0	0	0	0	0
OR-172	0	0	0	0	0	1
OR-173	0	0	0	0	1	1
OR-174	0	0	0	0	4	0
OR-175	0	0	21	19	28	19
OR-176	0	0	0	1	0	0
OR-177	0	0	0	0	1	0
OR-178	0	0	0	0	0	0
OR-179	0	0	0	0	0	0
OR-180	0	0	0	0	0	2
OR-181	0	0	1	0	0	0
OR-182	0	0	0	11	3	1
OR-183	0	0	0	20	21	0
OR-184	0	0	0	0	0	0
OR-185	0	0	0	0	0	0
OR-186	0	0	0	0	0	0
OR-187	0	0	0	0	0	1
OR-188	0	0	0	48	57	0
OR-190	0	0	0	0	0	0
OR-191	2	0	0	4	0	3
OR-192	0	0	1	0	0	0
OR-193	0	0	0	0	0	0
OR-194	0	0	0	0	0	0
OR-195	0	0	0	0	0	0
OR-196	0	0	0	0	0	0
OR-197	0	0	0	0	0	0
OR-198	0	0	1	0	0	0
OR-199	0	0	0	0	0	0
OR-200	0	0	0	0	0	0
OR-201	0	0	0	0	0	0
OR-202	0	0	0	0	0	0
OR-203	0	0	0	0	0	0
OR-204	0	0	0	0	0	0
OR-205	0	0	0	0	0	3
OR-206	0	0	1	0	0	2
OR-207	0	0	1	0	0	3
OR-208	0	0	0	0	0	3
OR-209	0	0	1	0	0	0
OR-210	0	0	0	0	0	0
OR-211	0	0	0	0	0	2
OR-212	0	0	0	0	0	0
OR-213	0	0	1	0	0	0
OR-214	0	0	0	0	0	1
OR-215	0	0	0	0	0	1
OR-216	0	0	0	0	0	0
OR-217	0	0	0	0	0	1
OR-218	0	0	0	0	0	0
OR-219	0	0	0	0	0	0
OR-220	0	0	0	0	0	0
OR-221	0	0	0	0	0	0
OR-222	0	0	0	0	0	0
OR-223	0	0	0	0	0	1
OR-224	0	0	0	0	0	0
OR-225	0	0	2	0	0	1
OR-226	0	0	1	0	0	1
OR-227	0	0	3	0	0	1
OR-228	0	0	0	0	0	0
OR-229	0	0	0	0	0	0
OR-230	0	0	0	0	0	0
OR-231	0	0	0	0	0	0
OR-232	0	0	1	0	0	0
OR-233	0	0	0	0	0	0
OR-234	0	0	0	0	0	0
OR-235	0	0	0	0	0	0
OR-236	0	0	11	0	0	19
OR-237	0	0	0	0	0	0
OR-238	0	0	0	0	0	0
OR-239	0	1	0	0	0	0
OR-240	0	0	0	0	0	0
OR-241	1	0	0	0	0	0
OR-242	0	0	0	0	0	0
OR-243	0	0	0	0	0	0
OR-244	0	0	2	0	0	1
OR-245	0	0	0	0	0	0
OR-246	0	1	1	0	1	3
OR-247	0	0	1	0	0	1
OR-248	0	0	1	0	0	1
OR-249	0	0	0	0	0	0
OR-250	0	0	0	0	0	2
OR-251	0	0	0	0	0	0
OR-252	0	0	2	0	0	1
OR-253	0	0	0	0	0	0
OR-254	0	0	0	0	0	0
OR-255	0	0	1	0	0	0
OR-256	0	0	0	2	5	0
OR-257	0	0	0	0	0	0
OR-258	0	0	0	0	0	2
OR-259	0	0	0	0	0	1
OR-260	0	0	0	0	0	0
OR-261	0	0	0	0	0	0
OR-262	0	0	0	0	0	0
OR-263	0	0	0	0	0	0
OR-264	0	0	0	0	0	0
OR-265	0	0	0	0	0	0
OR-266	0	0	0	0	0	0
OR-267	0	0	0	0	0	0
OR-268	0	0	0	0	0	1
OR-269	0	0	0	0	0	0
OR-270	0	0	0	0	0	0
OR-271	0	0	0	0	0	2
OR-272	0	0	0	0	0	0
OR-273	0	0	0	0	0	0
OR-274	0	0	0	0	0	0
OR-275	0	0	0	0	0	0
OR-276	0	0	0	0	0	1
OR-277	0	0	0	0	0	1
OR-278	0	0	0	0	0	1
OR-279	0	0	0	0	0	0
OR-280	0	0	0	0	0	1
OR-281	0	0	0	0	0	1
OR-282	0	0	0	0	0	0
OR-283	0	0	0	0	0	0
OR-284	0	0	0	0	0	0
OR-285	0	0	0	0	0	1
OR-286	0	0	2	0	0	6
OR-287	0	0	0	0	0	0
OR-288	0	0	0	0	0	0
OR-290	0	0	0	0	0	0
OR-291	0	0	0	0	0	0
OR-292	0	0	0	7	29	3
OR-293	0	0	0	0	0	0
OR-294	0	0	0	0	0	1
OR-295	0	0	0	0	0	0
OR-296	0	0	0	0	0	0
OR-297	0	1	0	0	0	0
OR-299	0	0	0	0	0	0
OR-300	0	0	0	0	0	0
OR-303	0	0	2	0	4	6
OR-304	0	0	0	0	2	0
OR-305	0	0	0	2	2	1
OR-306	0	1	0	5	0	5
OR-307	0	0	0	1	0	5
OR-308	0	0	0	1	0	0
OR-309	0	1	0	0	9	0
OR-310	0	0	0	0	0	0
OR-311	0	0	0	0	0	4
OR-312	0	0	0	0	0	0
OR-313	0	0	0	0	0	1
OR-314	0	0	0	0	0	0
OR-315	0	2	0	1	0	2
OR-316	0	0	0	0	0	3
OR-317	0	0	0	1	0	0
OR-318	0	0	1	0	0	1
OR-319	0	0	0	1	0	0
OR-320	0	0	0	0	0	0
OR-321	0	0	0	0	0	0
OR-322	0	0	0	0	0	0
OR-323	0	0	0	0	0	0
OR-324	0	0	0	1	0	0
OR-325	0	0	0	0	0	0
OR-326	0	0	0	0	0	0
OR-327	0	0	0	0	0	0
OR-328	0	0	3	2	1	0
OR-329	0	0	0	0	0	1
OR-330	0	0	1	1	0	2
OR-331	0	0	0	0	0	0
OR-332	0	0	0	0	0	0
OR-333	0	0	0	0	0	0
OR-335	0	0	0	0	0	0
OR-336	0	0	0	0	0	0
OR-337	0	0	2	0	0	0
OR-338	0	0	0	0	0	0
OR-340	0	0	0	0	0	0
OR-341	0	1	0	0	0	0
OR-342	0	0	0	0	0	1
OR-343	0	0	0	0	0	0
OR-345	0	0	0	1	0	0
OR-346	0	0	0	0	0	0
OR-347	0	0	0	0	0	0
OR-348	0	0	0	0	0	0
OR-349	0	0	0	0	0	0
OR-350	0	0	0	0	0	0
OR-351	0	0	0	0	0	0
OR-352	0	0	0	0	0	0
OR-353	0	6	7	2	11	8
OR-354	0	0	0	1	0	0
OR-355	0	0	0	0	0	1
OR-356	0	0	0	0	0	1
OR-357	0	0	0	0	0	0
OR-358	0	0	0	1	0	0
OR-359	0	0	0	2	0	0
OR-360	0	0	0	0	0	0
OR-361	0	0	0	0	0	0
OR-362	0	0	0	0	0	0
OR-363	0	0	0	0	0	0
OR-364	0	0	0	1	0	0
OR-365	0	0	0	0	0	0
OR-366	0	0	0	1	0	0
OR-367	0	0	3	0	0	0
OR-368	0	0	0	0	0	0
OR-369	0	0	0	0	0	0
OR-370	0	0	0	0	0	1
OR-371	0	0	0	0	0	0
OR-372	0	0	32	30	33	38
OR-373	0	0	0	3	0	0
OR-374	0	0	0	0	0	0
OR-375	0	0	0	0	0	0
OR-376	0	0	0	0	0	0
OR-377	0	1	0	0	0	0
OR-378	0	0	0	0	0	0
OR-379	0	0	0	0	0	0
OR-380	0	0	0	0	0	1
OR-382	0	0	1	0	0	0
OR-383	0	0	0	0	0	0
OR-384	0	0	0	0	0	0
OR-385	0	0	0	0	0	1
OR-386	0	0	0	0	0	0
OR-387	0	0	1	0	0	1
OR-388	0	0	0	0	0	0
OR-389	0	0	0	0	0	0
OR-390	0	0	0	0	0	0
OR-391	0	0	0	0	0	0
OR-392	0	0	0	0	0	0
OR-393	0	0	0	0	0	0
OR-394	0	0	0	0	0	1
OR-395	0	0	0	0	0	0
OR-396	0	0	0	0	0	0
OR-397	0	0	0	0	0	0
OR-398	0	0	0	0	0	0
OR-399	0	0	0	0	0	1
OR-400	0	0	0	0	0	2
OR-404	0	0	0	0	0	1
OR-405	0	0	0	1	0	0
OR-406	0	0	0	0	0	0
OR-407	0	0	0	0	2	0
OR-408	0	0	0	1	0	1
OR-409	0	0	0	1	0	0
OR-410	0	0	0	0	0	0
OR-411	0	0	0	0	0	0
OR-412	0	0	0	0	0	0
OR-413	0	0	0	0	0	0
OR-414	0	0	0	0	0	0
OR-415	0	0	0	0	0	0
OR-416	0	0	0	0	0	0
OR-417	0	0	0	0	0	0
OR-418	0	0	0	0	0	0
OR-419	0	0	0	1	0	0
OR-420	0	0	0	1	0	0
OR-421	0	0	0	1	0	0
OR-422	0	0	0	0	0	0
OR-423	0	0	0	0	0	1
OR-424	0	0	0	0	0	1
OR-425	0	0	0	0	0	0
OR-426	0	0	0	0	0	0
OR-427	0	0	0	0	0	0
OR-428	0	0	0	0	0	0
OR-429	0	0	0	2	0	0
OR-430	0	0	0	0	0	0
OR-431	0	0	0	0	0	0
OR-432	0	0	0	0	0	0
OR-433	0	0	0	1	0	0
OR-434	0	0	0	0	0	0
OR-435	0	0	0	1	0	0
OR-436	0	0	0	3	1	0
OR-437	0	0	0	0	0	0
OR-438	0	0	0	1	0	0
OR-439	0	0	0	0	0	0
OR-440	0	0	0	1	0	0
OR-442	0	0	0	0	0	1
OR-443	0	0	0	0	0	0
OR-444	0	0	0	0	0	0
OR-445	0	0	0	1	0	0
OR-446	0	0	0	0	0	0
OR-447	0	0	0	1	0	0
OR-448	0	0	0	0	0	0
OR-449	0	0	0	0	0	0
OR-450	0	0	0	1	1	0
OR-451	0	0	0	0	1	0
OR-452	0	0	0	0	0	0
OR-453	0	0	0	0	0	0
OR-454	0	0	0	0	0	0
OR-455	0	0	0	0	1	0
OR-456	0	0	0	0	0	0
OR-457	0	0	0	0	1	0
OR-458	0	0	0	0	0	0
OR-459	1	1	0	2	1	0
OR-460	0	0	0	1	0	1
OR-461	0	0	1	1	8	0
OR-462	0	0	0	1	0	0
OR-463	0	0	0	0	0	1
OR-464	0	0	0	0	0	2
OR-465	0	0	0	0	1	0
OR-466	0	0	0	0	0	0
OR-467	0	0	0	2	1	0
OR-468	0	0	0	0	0	0
OR-469	0	0	1	2	0	0
OR-470	0	0	0	0	0	0
OR-471	0	0	0	0	0	0
OR-472	0	0	0	0	0	0
OR-473	0	0	0	0	0	0
OR-474	0	0	0	0	0	0
OR-475	0	0	0	0	0	0
OR-476	0	0	0	0	0	0
OR-477	0	0	0	0	0	1
OR-478	0	0	0	0	0	0
OR-479	0	0	0	0	0	0
OR-480	0	0	0	0	0	0
OR-481	0	0	0	0	0	0
OR-482	0	0	0	0	0	0
OR-483	0	0	0	0	1	0
OR-484	0	0	1	0	0	0
OR-485	0	0	0	0	0	0
OR-486	0	0	0	0	0	1
OR-487	0	0	1	0	0	1
OR-488	0	0	0	0	0	0
OR-489	0	0	0	0	0	0
OR-490	0	0	0	0	0	0
OR-491	0	0	0	0	0	0
OR-492	0	0	1	0	0	0
OR-493	0	0	0	0	0	0
OR-494	0	0	0	0	0	1
OR-495	0	0	0	0	0	0
OR-496	0	0	1	0	0	1
OR-497	0	0	0	0	0	2
OR-498	0	0	0	0	0	1
OR-499	0	0	0	0	0	0
OR-500	0	0	0	1	0	0
OR-501	0	0	0	0	0	0
OR-502	0	0	0	0	0	1
OR-503	0	0	0	0	0	1
OR-504	1	1	0	2	2	1
OR-505	0	0	0	0	0	0
OR-506	0	0	0	0	0	0
OR-507	0	0	0	0	0	0
OR-508	0	0	0	0	0	0
OR-509	0	0	0	0	0	0
OR-510	0	0	0	0	0	0
OR-511	0	0	0	0	0	0
OR-512	0	0	0	0	0	0
OR-513	0	0	0	0	0	1
OR-514	0	0	0	0	0	0
OR-515	0	0	0	0	0	0
OR-516	0	0	0	0	0	0
OR-517	0	0	0	0	0	0
OR-518	0	0	0	0	0	2
OR-519	0	0	0	0	0	1
OR-520	0	0	0	0	0	0
OR-521	0	0	0	0	0	0
OR-522	0	0	0	0	0	1
OR-523	0	0	0	0	0	0
OR-524	0	0	0	0	0	1
OR-525	0	0	0	0	0	0
OR-526	0	0	0	0	0	2
OR-527	0	0	0	0	0	1
OR-528	0	0	0	0	0	0
OR-529	0	0	0	0	0	0

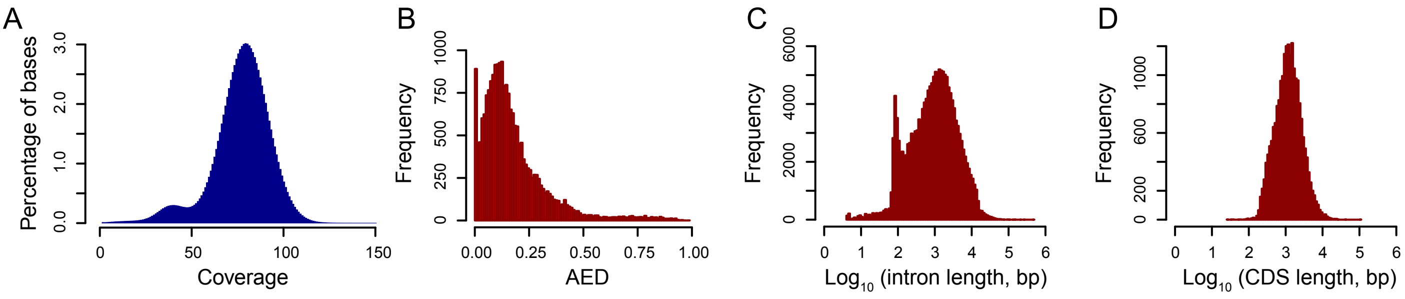

Figure S1. Distributions showing the main features of the Bloom filter-based genome assembly of the Pyrenean desman and the predicted protein-coding genes. (A) Coverage of the short-insert sequencing data. (B) Annotation edit distances (AED) of the predicted genes. (C) Logarithm of intron length of the predicted genes. (D) Logarithm of coding sequence (CDS) length of the predicted genes.

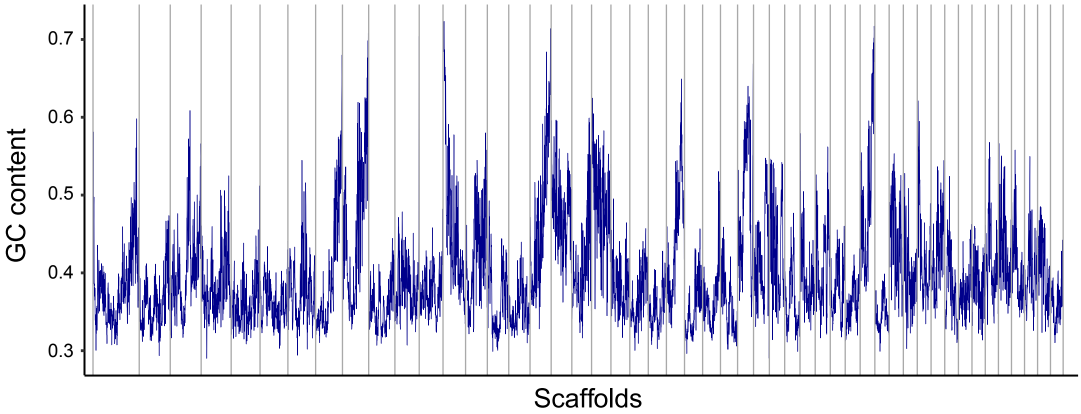

Figure S2. GC content variation in the autosomal scaffolds longer than 10 Mb.

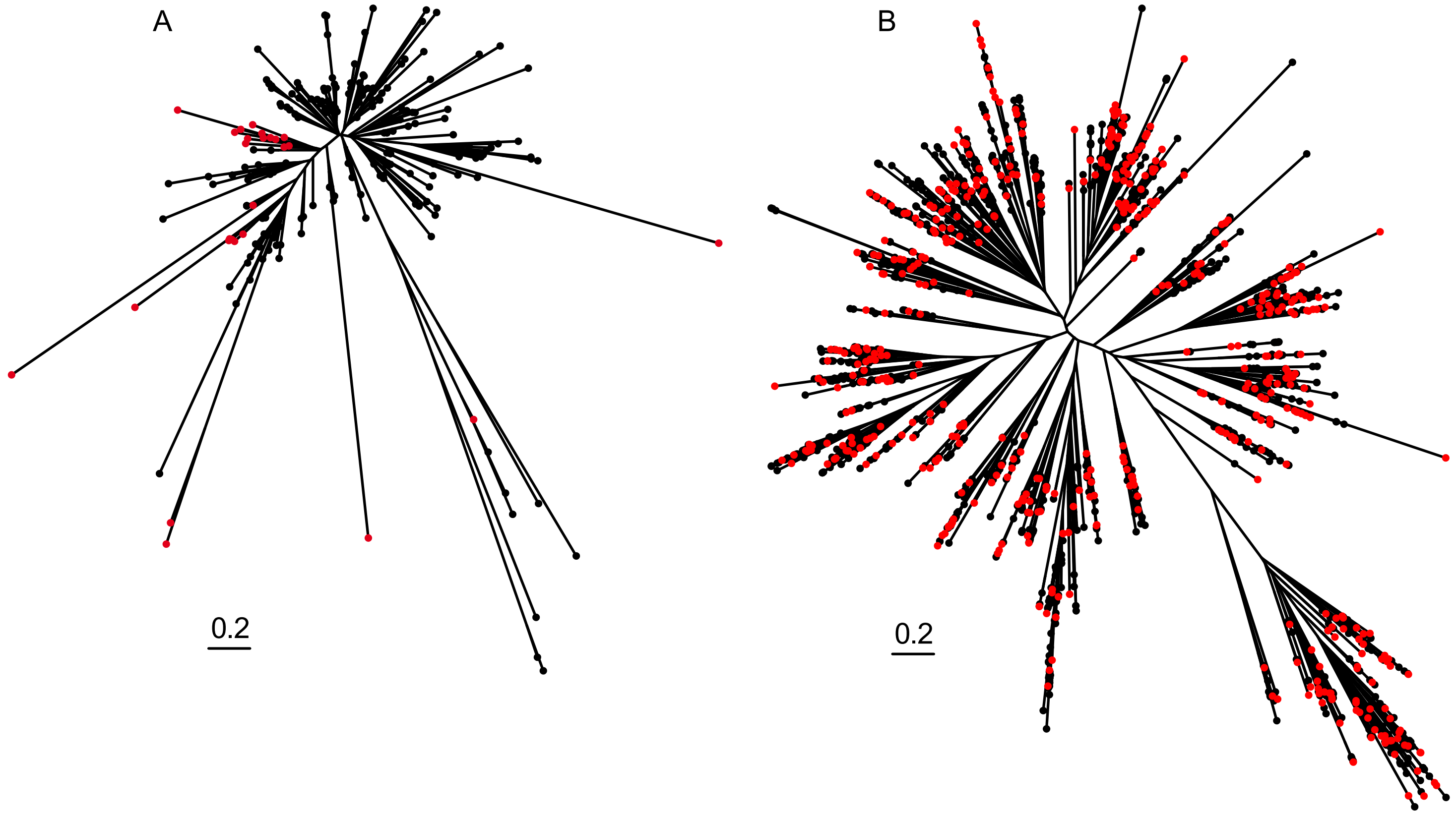

Figure S3. Maximum-likelihood phylogenetic trees constructed from the amino acid alignments of (A) 26 Pyrenean desman MHC-I genes together with those of the mole *Condylura cristata*, human and several mammals from Abduriyim et al. 2019, and (B) 529 Pyrenean desman olfactory receptor genes together with those of *Condylura cristata* and human. The Pyrenean desman sequences are shown with a red circle and those of other mammals with a black circle. The scale represents 0.2 substitutions per position and is the same in both cases.

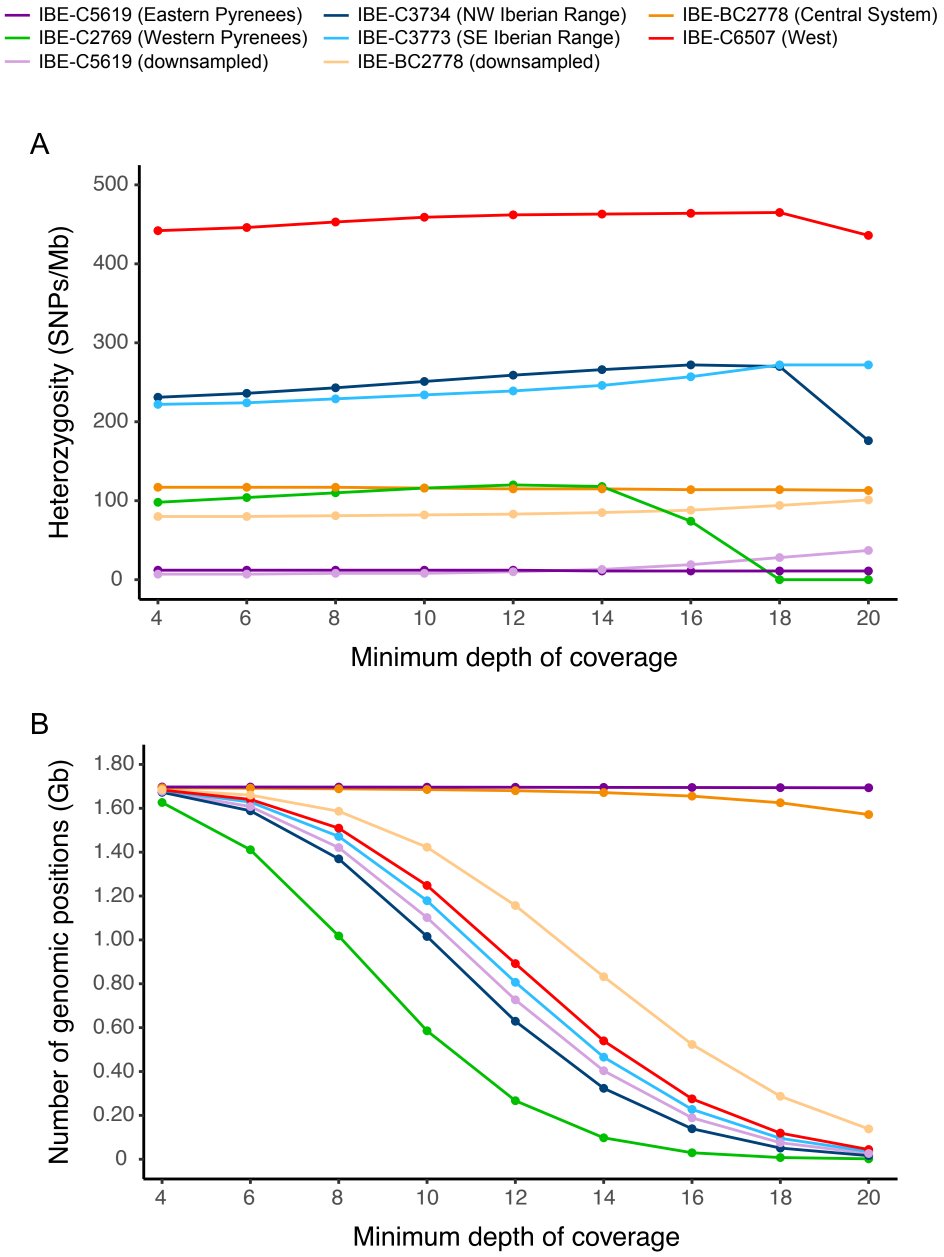

Figure S4. (A) Genome-wide heterozygosity rate and (B) number of genomic positions passing quality filters for different values of minimum depth of coverage for SNP calling.

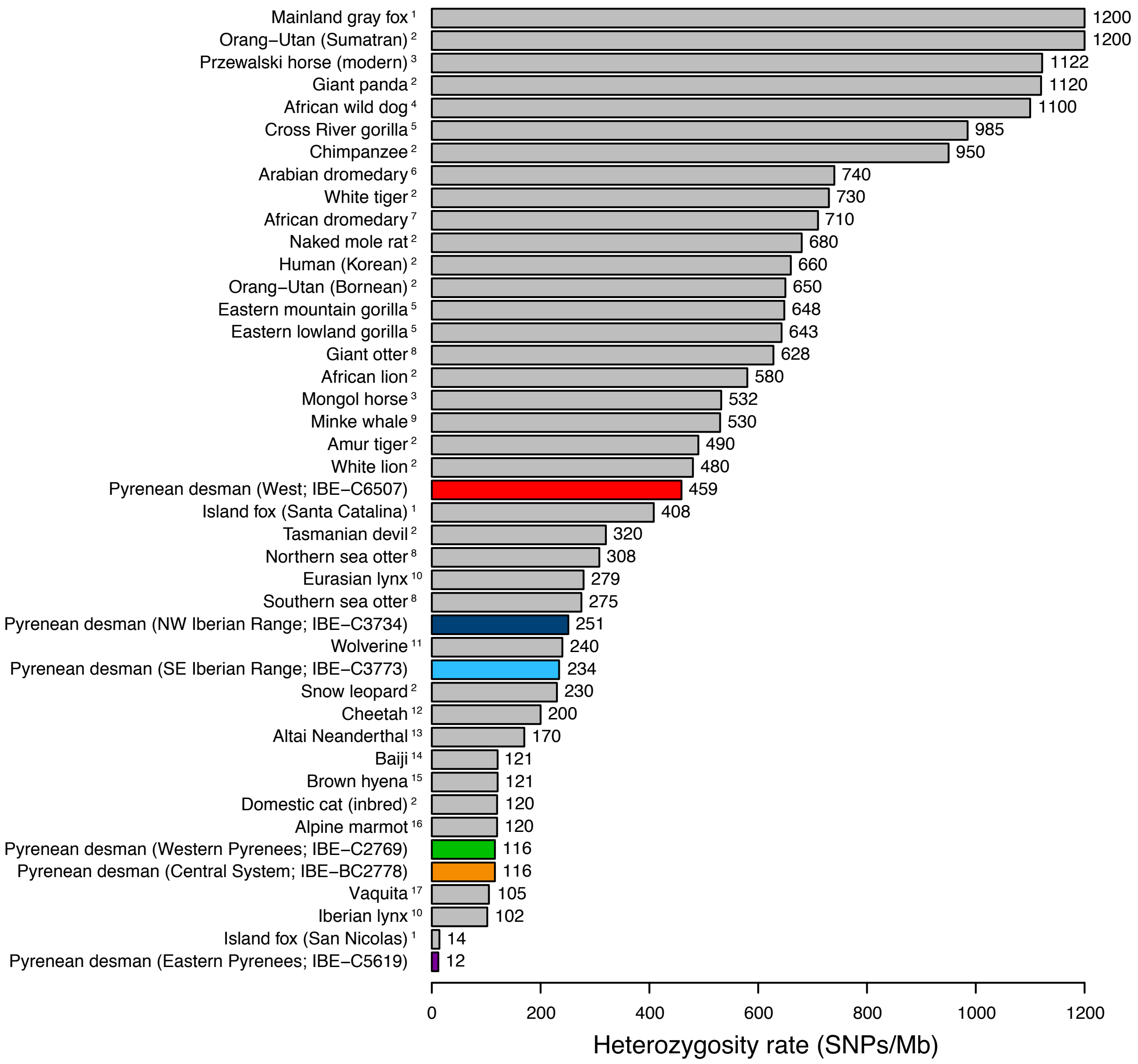

[1] Robinson, J. A. et al. Genomic Flatlining in the Endangered Island Fox. Curr. Biol. 26, 1183–1189 (2016).
[2] Cho, Y. S. et al. The tiger genome and comparative analysis with lion and snow leopard genomes. Nat. Commun. 4, (2013).
[3] Der Sarkissian, C. et al. Evolutionary genomics and conservation of the endangered Przewalski's horse. Curr. Biol. 25, 2577–2583 (2015).
[4] Armstrong, E. E. et al. Cost-effective assembly of the African wild dog (Lycaon pictus) genome using linked reads. Gigascience 8, 1–10 (2019).
[5] Xue, Y. et al. Mountain gorilla genomes reveal the impact of long-term population decline and inbreeding. Science (80-.). 348, 242–245 (2015).
[6] Wu, H. et al. Camelid genomes reveal evolution and adaptation to desert environments. Nat. Commun. 5, (2014).
[7] Fitak, R. R., Mohandesan, E., Corander, J. & Burger, P. A. The de novo genome assembly and annotation of a female domestic dromedary of North African origin. Mol. Ecol. Resour. 16, 314–324 (2016).
[8] Beichman, A. C. et al. Aquatic Adaptation and Depleted Diversity: A Deep Dive into the Genomes of the Sea Otter and Giant Otter. Mol. Biol. Evol. 36, 2631–2655 (2019).
[9] Yim, H. S. et al. Minke whale genome and aquatic adaptation in cetaceans. Nat. Genet. 46, 88–92 (2014).
[10] Abascal, F. et al. Extreme genomic erosion after recurrent demographic bottlenecks in the highly endangered Iberian lynx. Genome Biol. 17, 251 (2016).
[11] Ekblom, R. et al. Genome sequencing and conservation genomics in the Scandinavian wolverine population. Conserv. Biol. 32, 1301–1312 (2018).
[12] Dobrynin, P. et al. Genomic legacy of the African cheetah, Acinonyx jubatus. Genome Biol. 16, 1–19 (2015).
[13] Prüfer, K. et al. The complete genome sequence of a Neanderthal from the Altai Mountains. Nature 505, 43–49 (2014).
[14] Zhou, X. et al. Baiji genomes reveal low genetic variability and new insights into secondary aquatic adaptations. Nat. Commun. 4, 1–6 (2013).
[15] Westbury, M. V. et al. Extended and continuous decline in effective population size results in low genomic diversity in the world's rarest hyena species, the brown Hyena. Mol. Biol. Evol. 35, 1225–1237 (2018).
[16] Gossmann, T. I. et al. Ice-Age Climate Adaptations Trap the Alpine Marmot in a State of Low Genetic Diversity. Curr. Biol. 29, 1712–1720.e7 (2019).
[17] Morin, P. A. et al. Reference genome and demographic history of the most endangered marine mammal, the vaquita. Mol. Ecol. Resour. 00, 1-13. (2020).

Figure S5. Genome-wide heterozygosity rate for different mammalian species, most of them of conservation concern. Values of different Pyrenean desmans sequenced in this work are shown in color.

A

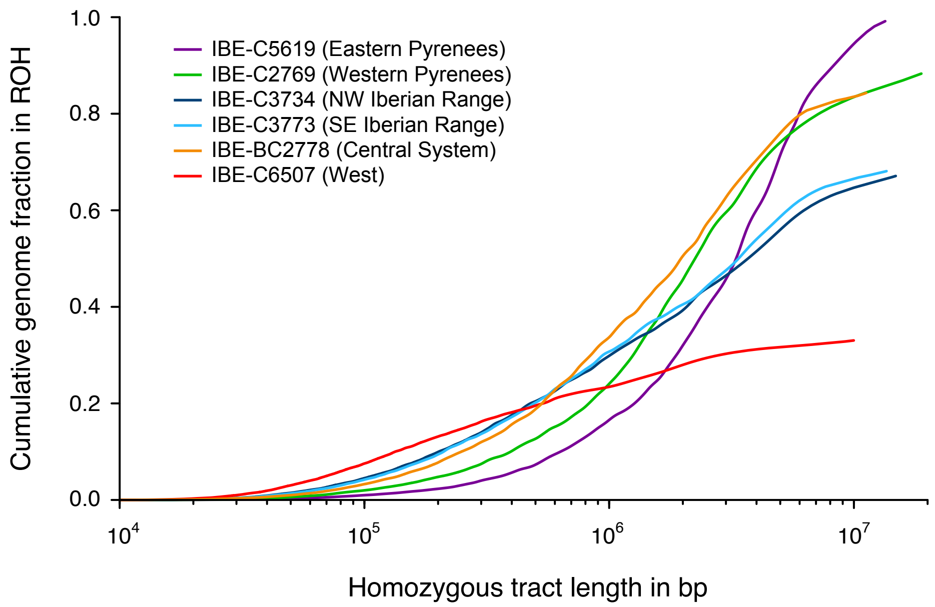

B

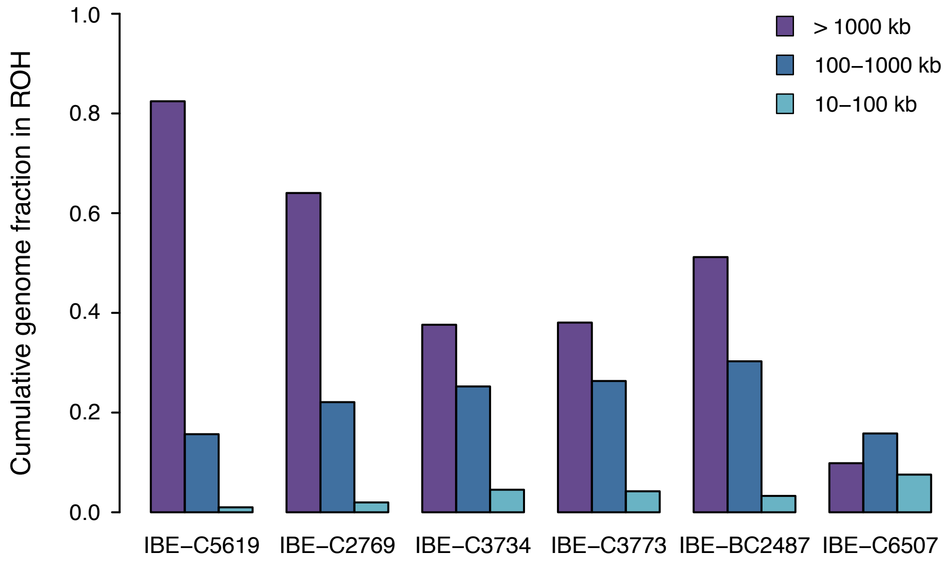

Figure S6. (A) Cumulative proportion of the genome contained in ROH below the length displayed on the X axis in each individual. (B) Cumulative proportion of the genome in ROH for different size classes in each individual.

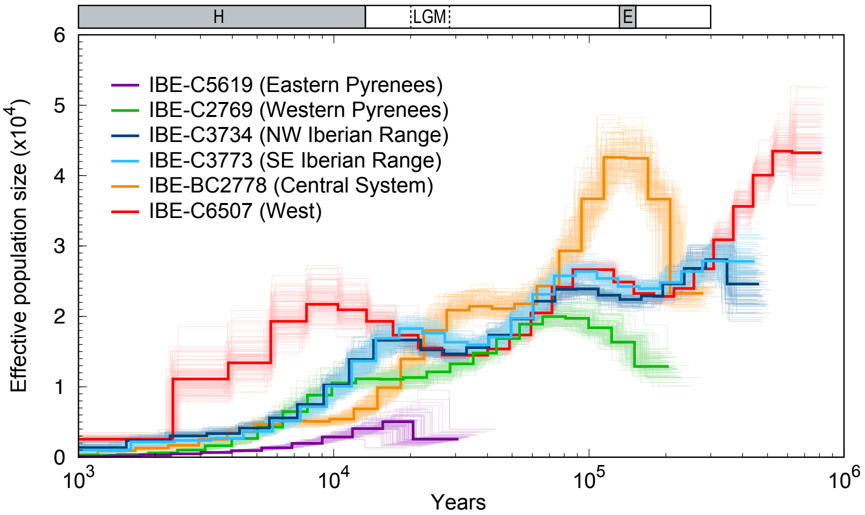

Figure S7. Historical effective population size inferred from the Pyrenean desman genomes by PSMC. The lighter colored lines of the same color represent the 100 bootstrap replicates. The result is scaled with a mutation rate (μ) of 5 x 10^{-9} mutations/site/generation and an average generation time of 2 years. The last two interglacial periods, Holocene (H) and Eemian (E), are indicated with grey boxes and the Last Glacial Maximum (LGM) with dashed lines.

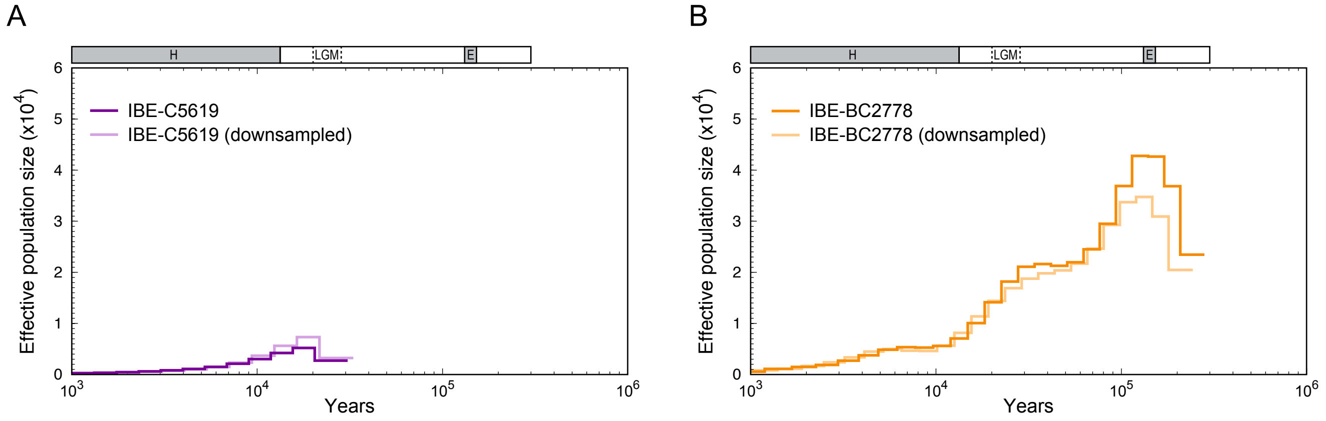

Figure S8. Historical effective population size inferred from the Pyrenean desman downsampled genomes by PSMC for individuals (A) IBE-C5619 and (B) IBE-BC2778. The result is scaled with a mutation rate (μ) of 5 x 10^{-9} mutations/site/generation and an average generation time of 2 years. The last two interglacial periods, Holocene (H) and Eemian (E), are indicated with grey boxes and the Last Glacial Maximum (LGM) with dashed lines.
